# Association of frailty with adverse outcomes in surgically treated geriatric patients with hip fracture: A meta-analysis and trial sequential analysis

**DOI:** 10.1371/journal.pone.0305706

**Published:** 2024-06-21

**Authors:** Xiaomeng Dong, Xiuguo Zhang, Fang Hu, Shuhong Yang, Zengchao Hong, Qian Geng

**Affiliations:** 1 Department of 2^nd^ Operating Room, Hebei Medical University Third Hospital, Shijiazhuang, Hebei, China; 2 Department of Nursing, Hebei Medical University Third Hospital, Shijiazhuang, Hebei, China; Instituto Nacional de Geriatria, MEXICO

## Abstract

**Objective:**

Some studies have associated frailty and prognostic outcomes in geriatric hip fracture patients, but whether frailty can predict postoperative outcomes remains controversial. This review aims to assess the relationship between frailty and adverse postoperative outcomes in geriatric patients with hip fracture.

**Methods:**

Based on electronic databases, including PubMed, Embase, Web of Science, Cumulative Index to Nursing and Allied Health Literature, the Cochrane Library, Chinese National Knowledge Infrastructure, and WanFang Data, we systematically searched for studies that investigated the association between frailty and adverse outcomes among patients aged 60 or over after hip fracture surgery. Stata 17.0 and Trial Sequential Analysis viewer software were used to obtain pooled estimates and verify whether the sample size was sufficient and the evidence robust.

**Results:**

Twenty-one studies involving 49,196 patients were included for quantitative analysis. Compared with nonfrail patients, frail patients had a higher risk of inpatient mortality (risk ratio [RR] = 1.93, 95% confidence interval [CI]: 1.66–2.23), 30-day mortality (RR = 2.13, 95% CI: 1.23–3.70), and 1-year mortality (RR = 2.44, 95% CI: 1.47–4.04). Frailty can significantly predict postoperative complications (RR = 1.76, 95% CI: 1.38–2.23), including delirium, pneumonia, cardiac complications, urinary tract infection, and surgical site infection; the association between frailty and deep venous thrombosis/pulmonary embolism and acute kidney injury needs further analysis. Trial sequential analysis showed that the findings regarding mortality were reliable and robust.

**Conclusion:**

This meta-analysis provides detailed information indicating that frailty is a substantial predictor of mortality and selected postoperative complications.

## Introduction

Defined as a fracture occurring in the area between the edge of the femoral head and 5 cm below the lesser trochanter, hip fracture (HF) is now considered an important public health problem and is associated with increased mortality and adverse outcomes in the older population [1, 2]. Approximately 1.6–2 million HFs occur per year [3], and more than 95% of HFs occur in patients aged 60 and over [4]. With a rapidly aging population, the number of HF cases in older adults is predicted to be 4.5 million in 2050, with cases in Asia, particularly China, accounting for more than 50% [5]. China is one of the most populous countries, and nearly 17.9% of the population was aged 60 years or over in 2018 [6, 7]. Such a high incidence of HF in older people place a substantial burden on one’s family and the whole health care system. Many older patients undergoing surgery may suffer more negative survival outcomes and postoperative outcomes than younger patients [8]. Previous studies have reported that HF, the most devastating result of osteoporosis, is likely to contribute to serious disability, excess mortality, increased healthcare expenditures [9] and socioeconomic burdens [10–13]. Thus, there is an urgent need to assess the risk of HF surgery, especially in older patients.

Frailty, an age-associated biological syndrome, results from a decline in reserve and resistance to stressors across multiple physiologic and organ systems [14, 15]. With the growth of the global aging population, frailty is increasingly associated with the postoperative prognosis of geriatric HF patients. Recently, literature that offers contradictory findings about whether frailty can significantly predict postoperative mortality has emerged. Sang et al. [16] found that there was no link between frailty and inpatient mortality. Existing meta-analyses [1, 17, 18] recognize that frailty is associated with mortality and inpatient complications; however, these articles only mention total complications with rough data rather than mentioning specific complications, including delirium, pneumonia, and pulmonary embolism. Additionally, previous studies are limited in quality, and many have small sample sizes. Given the adverse postoperative outcomes associated with frailty in older individuals, the associations of frailty with postoperative mortality and complications need to be addressed.

The hypothesized associations of frailty with postoperative mortality and complications in older HF patients await rigorous testing. We performed this meta-analysis to investigate whether frailty was associated with postoperative mortality and complications among older HF patients; we also conducted a trial sequential analysis (TSA) to evaluate the potency of the results.

## Methods

The study protocol has been registered at PROSPERO (CRD42023448370). This meta-analysis was conducted in accordance with the Preferred Reporting Items for Systematic Reviews and Meta-Analyses (PRISMA) 2020 reporting statement [19] (S1 Table).

### Search strategy

Two authors (XMD, FH) independently searched seven electronic databases from inception to 21:00 on 23 June 2023: PubMed, Embase, Web of Science, Cumulative Index to Nursing and Allied Health Literature (CINAHL, through EBSCOhost), the Cochrane Library, Chinese National Knowledge Infrastructure (CNKI), and WanFang Data. The language was restricted to English and Chinese. Additionally, we manually screened the reference lists of studies to identify additional papers to ensure that all available studies were included. The detailed search queries for PubMed are listed in S2 Table.

### Inclusion and exclusion criteria

Studies were included if they met the following criteria: 1) they were cohort studies, cross-sectional and case‒control studies reporting all-cause mortality and surgical complications; 2) study participants were aged 60 years or older and undergoing HF surgery; 3) frailty was considered a major exposure; 4) frail groups and nonfrail groups were clearly categorized by validated assessment instruments for frailty; and 5) outcomes regarding the risk ratio (RR) or hazard ratio (HR) with a 95% confidence interval (CI) were displayed or sufficient information was provided for calculating these data. Studies were excluded if they met any of the following criteria: 1) they were qualitative studies, conference abstracts, case reports, reviews, study protocols; 2) frailty status was measured by other evaluation methods or one marker of frailty (e.g., low hand grip strength or gait speed); or 3) poor outcomes reported by original studies were influenced by the frailty status and combinations of other factors.

### Study selection process

After comprehensively searching for potentially eligible studies, two reviewers (XMD and FH) screened selected titles and abstracts to find relevant articles. Selected papers were retrieved to evaluate whether these potentially eligible studies satisfied the inclusion criteria. If a consensus on final inclusion of a study could not be reached, a third author (ZCH) helped resolve the disagreement.

### Data extraction

Two authors separately retrieved the following data from accepted studies: first author’s name, year of publication, study design, country, setting, period, sample size, patient characteristics (mean or median age, sex proportion), frailty assessment tool used, prevalence of frailty measured, and postoperative outcomes in relation to frailty and HF groups. A third author (ZCH) rendered the decision when disagreements of extracted content arose.

### Quality assessment and risk of bias

We used the Newcastle‒Ottawa Scale (NOS) to independently assess the risk of bias of cohort studies [20]. This scale contains eight items that are divided into three domains, including selection, comparability, and ascertainment of outcomes or exposure [21]. The score ranges from 0 to 9 points; the more stars an included study receives, the higher its quality. The quality of studies was classified as follows: high (8–9 stars), moderate (5–7 stars), or poor (0–4 stars) [22]. Studies with a score of 0–4 stars were excluded from this review due to their high risk of bias.

### Statistical analysis

The meta-analysis was performed using STATA software. We used the summary RRs and corresponding 95% CIs of the included studies as the effect measures to assess the association of frailty with postoperative outcomes. The statistical heterogeneity between studies was evaluated by Cochrane’s *Q* test and *I*^2^ value. When the *Q* test had a 2-tailed *P*<0.10 and *I*^2^≥50%, we considered the heterogeneity to be significant [22, 23] and adopted a random-effects model to pool all data. Additionally, when the *Q* test had a 2-tailed *P≥*0.10 and *I*^2^<50%, indicating the existence of homogeneity, a fixed-effects model was selected for synthesizing all data. To explore the potential source of heterogeneity, univariate meta-regression analysis was performed, and we conducted three prespecified subgroup analyses according to geographic region, study design (retrospective cohort study vs. prospective cohort study), and frailty assessment tool used (Frailty Index [FI] and its modified versions vs. Clinical Frailty Scale [CFS] vs. other frailty instruments). Meta-regression results with a 2-tailed *P*<0.05 were considered significant [24]. Subgroup analyses were performed if there were at least two studies in each subgroup category [25]. We performed sensitivity analysis by omitting each study separately to assess the stability of the results. Egger’s tests were used to detect publication bias. Funnel plots were also generated to evaluate publication bias if there were at least 10 studies in each outcome measure group [26]. In this meta-analysis, a 2-tailed *P*<0.05 was defined as statistically significant.

TSA was performed to evaluate the type I and type II errors in a meta-analysis of studies with a limited sample size to further examine whether the current evidence was conclusive and adequately reliable [27, 28]. Data were analyzed using TSA viewer software. In TSA, the required information size (RIS), monitoring, and futility boundaries were computed based on the following parameters: prespecified type I error of 5% (2-sided) and type II error of 20% (power at 80%), relative risk reduction (RRR) evaluated by [RRR = 100% ×(1-RR)], and incidence in the control arm set by the pooled frail group event proportions from the total frail group. If the cumulative Z-curve surpassed the trial sequential monitoring boundary and the RIS boundary, no further trials were needed, the conclusion drawn in this meta-analysis was positive, and the evidence was considered robust [29]; otherwise, more large-scale trials were required to verify the consistency of the evidence.

## Results

### Search results

**Fig 1:**
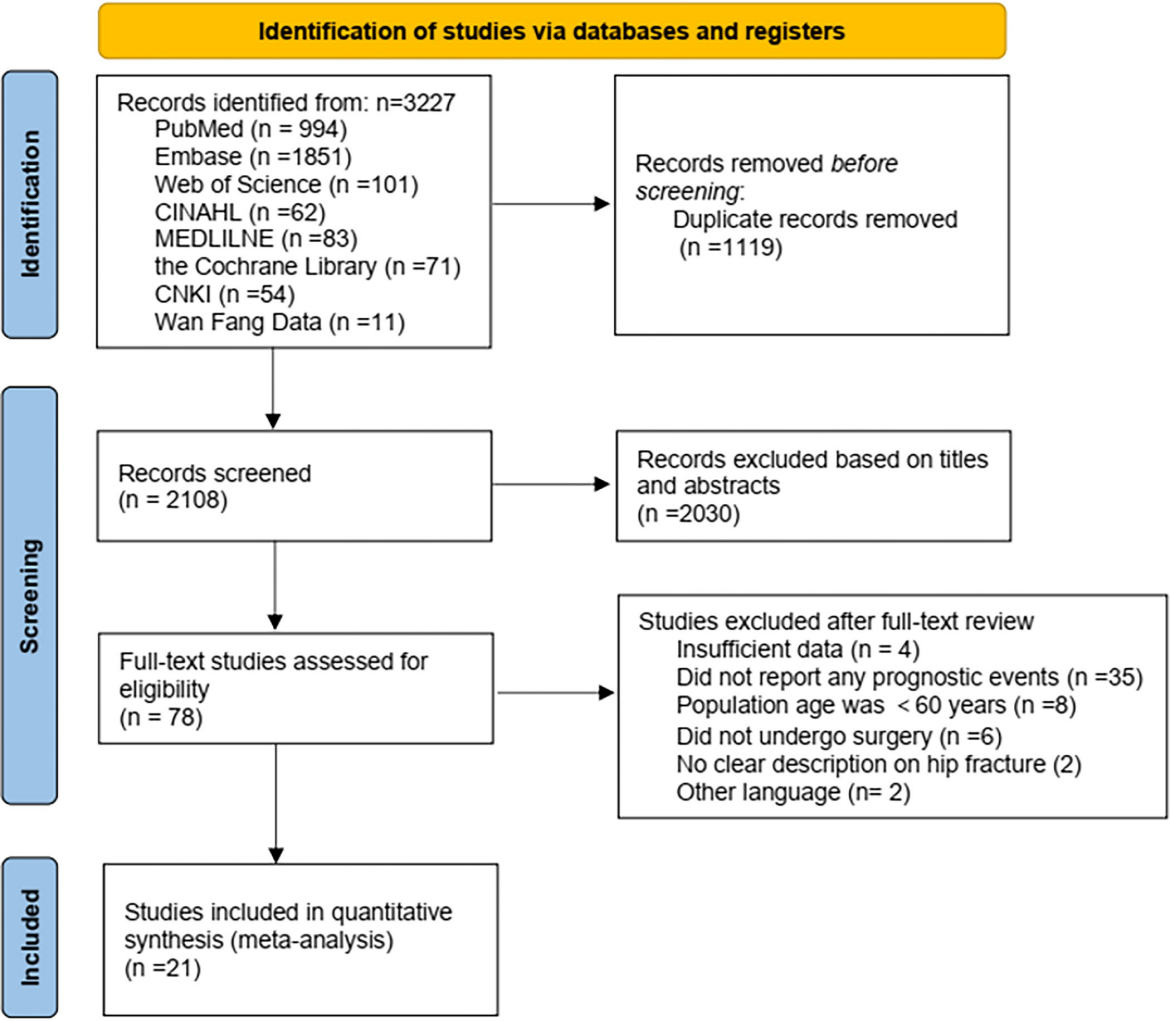
PRISMA flow diagram for the study selection process.

A total of 3,227 studies were retrieved, as shown in Fig 1. After the removal of duplicates, we examined the titles and abstracts. 57 articles were excluded for different reasons by careful full-text assessment. Finally, 21 articles [13, 16, 30–48] met the inclusion and exclusion criteria.

### Study characteristics

Twenty-one studies [13, 16, 30–48] involving 49,196 participants reported the association between frailty and postoperative outcomes among geriatric HF patients. The studies were performed from 2003 to 2022. Most participants in this meta-analysis were 65 years and older. Thirteen retrospective cohort studies [13, 16, 30, 33, 34, 36, 37, 40, 42–46] and 8 prospective cohort studies [31, 32, 35, 38, 39, 41, 47, 48] were conducted in China (n = 6) [13, 40, 43, 46–48], the United States (n = 4) [16, 30, 31, 34], Italy (n = 2) [38, 39], Singapore (n = 2) [32, 45], Australia (n = 2) [36, 37], the United Kingdom (n = 1) [41], the Netherlands (n = 1) [35], Korea (n = 1) [33], Japan (n = 1) [42], and India (n = 1) [44]. Fourteen studies [13, 16, 30, 34–37, 39, 41–46] reported on all-cause mortality. Eight studies focused on postoperative complication [31–34, 40, 43, 44, 46]. Multiple frailty assessment tools were utilized in this meta-analysis, including FI or modified Frailty Index (mFI) proposed by Rockwood *et al*. or the chart-derived Frailty index (CFI) reported by Amrock [13, 30, 36, 38–40, 43, 47], FRAIL Scale (FS) [34, 48], CFS [37, 41, 44] or Rock Frailty Score (RFS) [16], Modified Fried Frailty Index (mFFI) [31], Reported Edmonton Frailty Scale (REFS) [32], Hospital Frailty Risk Score (HFRS) [42, 45], Veiligheids Management Systeem (VMS) Frailty Score [35], Laboratory Frailty Index (FI-lab) [46], and Hip-Multidimensional Frailty Score (Hip-MFS) [33]. The characteristics of the included studies are summarized in S3 Table.

### Risk-of-bias assessment

In this meta-analysis, the mean (range) quality score of the identified studies was 8.19 stars (7–9 stars). Multivariate regression analysis was conducted in 11 studies [16, 30, 32, 35, 38, 39, 42–46] to control for the most important variables, such as age, sex, type of fracture, and type of anesthesia. Overall, the 21 included studies were of high quality (shown in S4 Table).

### Mortality predicted by frailty

**Fig 2:**
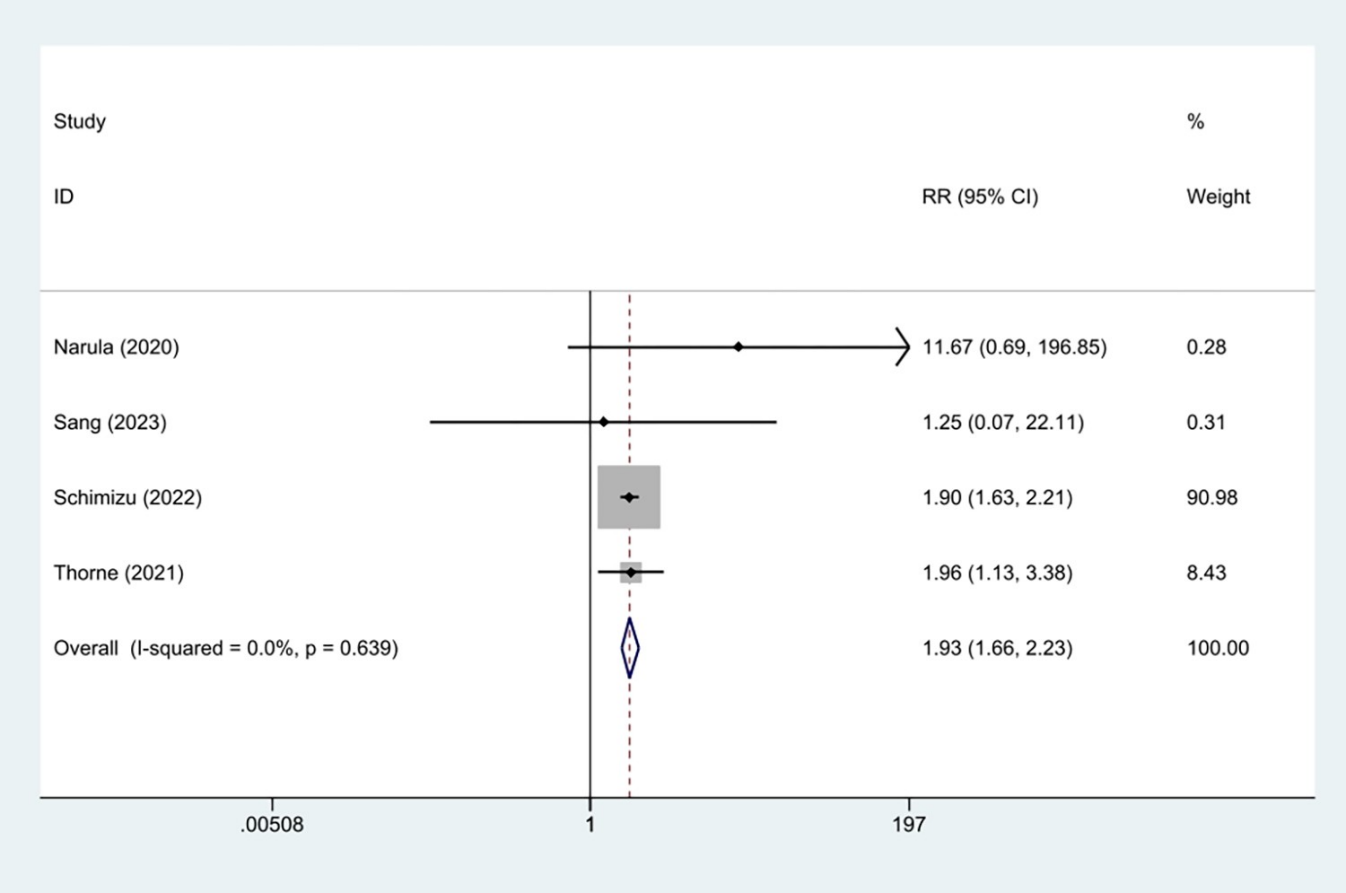
Forest plot of the RRs for the association between frailty and inpatient mortality.

**Fig 3:**
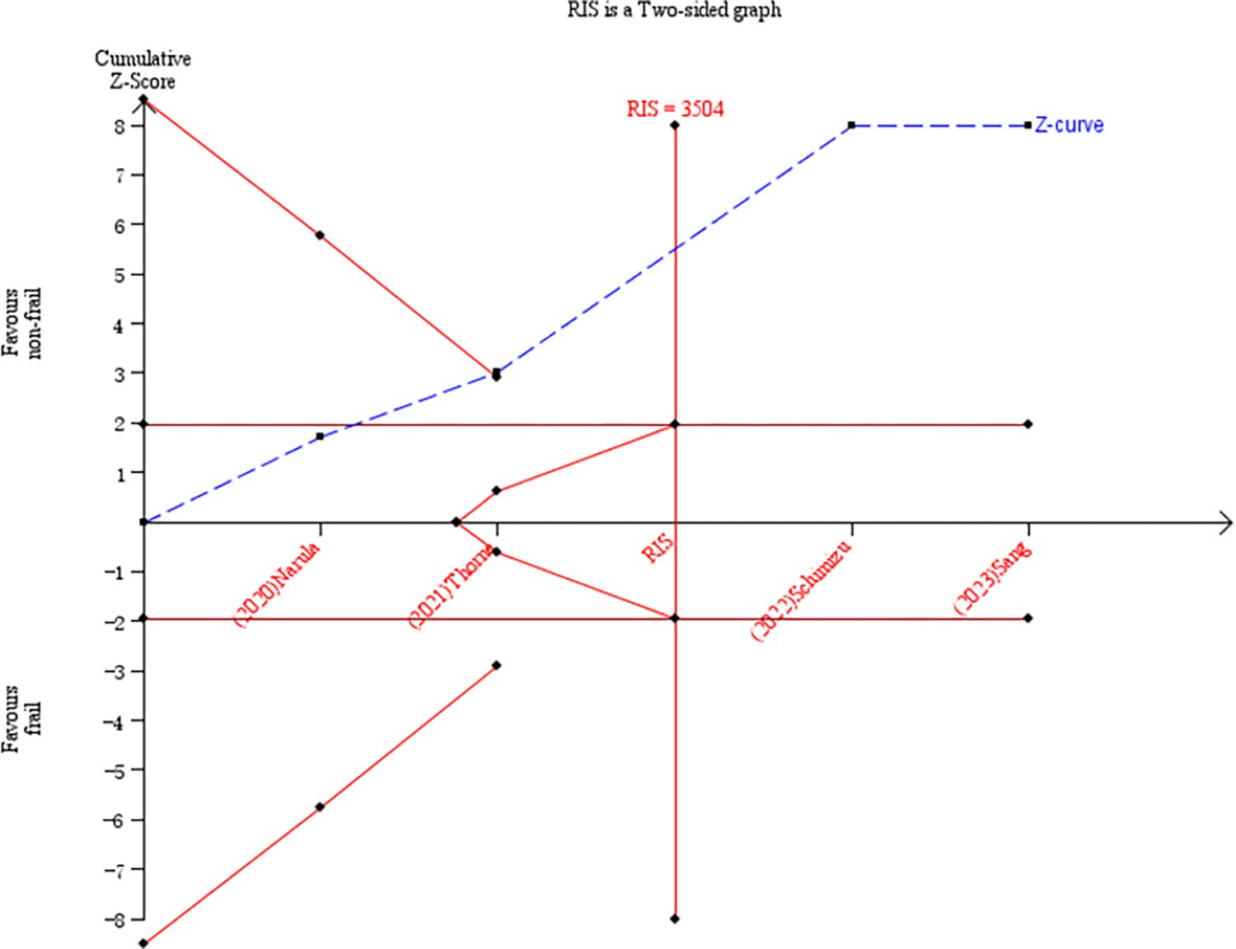
TSA of inpatient mortality predicted by frailty.

Among all included studies, the association of frailty with mortality of different durations was explored in 14 papers, including inpatient mortality, 30-day mortality, and 1-year mortality. Four studies were included in a meta-analysis to explore the relationship between frailty and inpatient mortality in HF patients [16, 37, 41, 42]. Using crude data with nonfrail patients as the control, the frail group displayed a significantly higher risk of inpatient mortality (RR = 1.93, 95% CI: 1.66–2.23 *I*^2^ = 0%, *P*<0.001; Fig 2). TSA was used to calculate the RIS. In the TSA, the risks of type I and type II errors were set to 5% and 20%, respectively. We defined the RRR and incidence in the frail group as 49.0% and 3.05%, respectively. In this TSA, the Z-curve crossed the trial sequential monitoring boundary and conventional benefit boundary. We found that the current sample size (38,470) reached the RIS of 3,504, further demonstrating that no more studies were needed for a stable conclusion in this meta-analysis (Fig 3).

**Fig 4:**
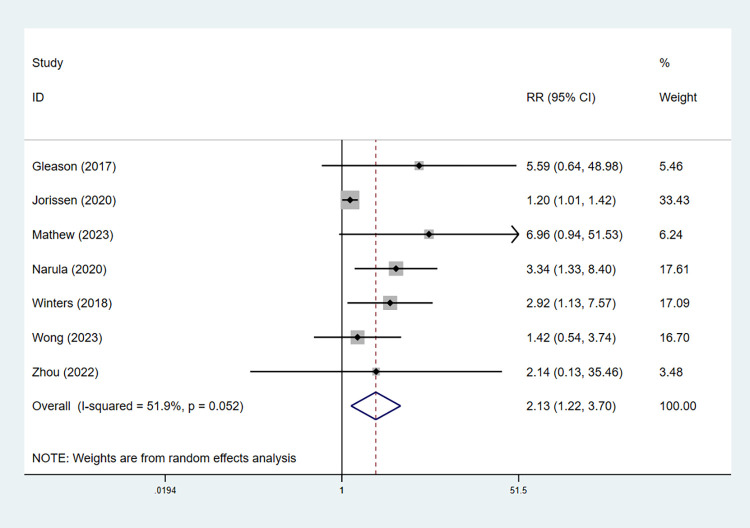
Forest plot of the RRs for the association between frailty and 30-day mortality.

**Fig 5:**
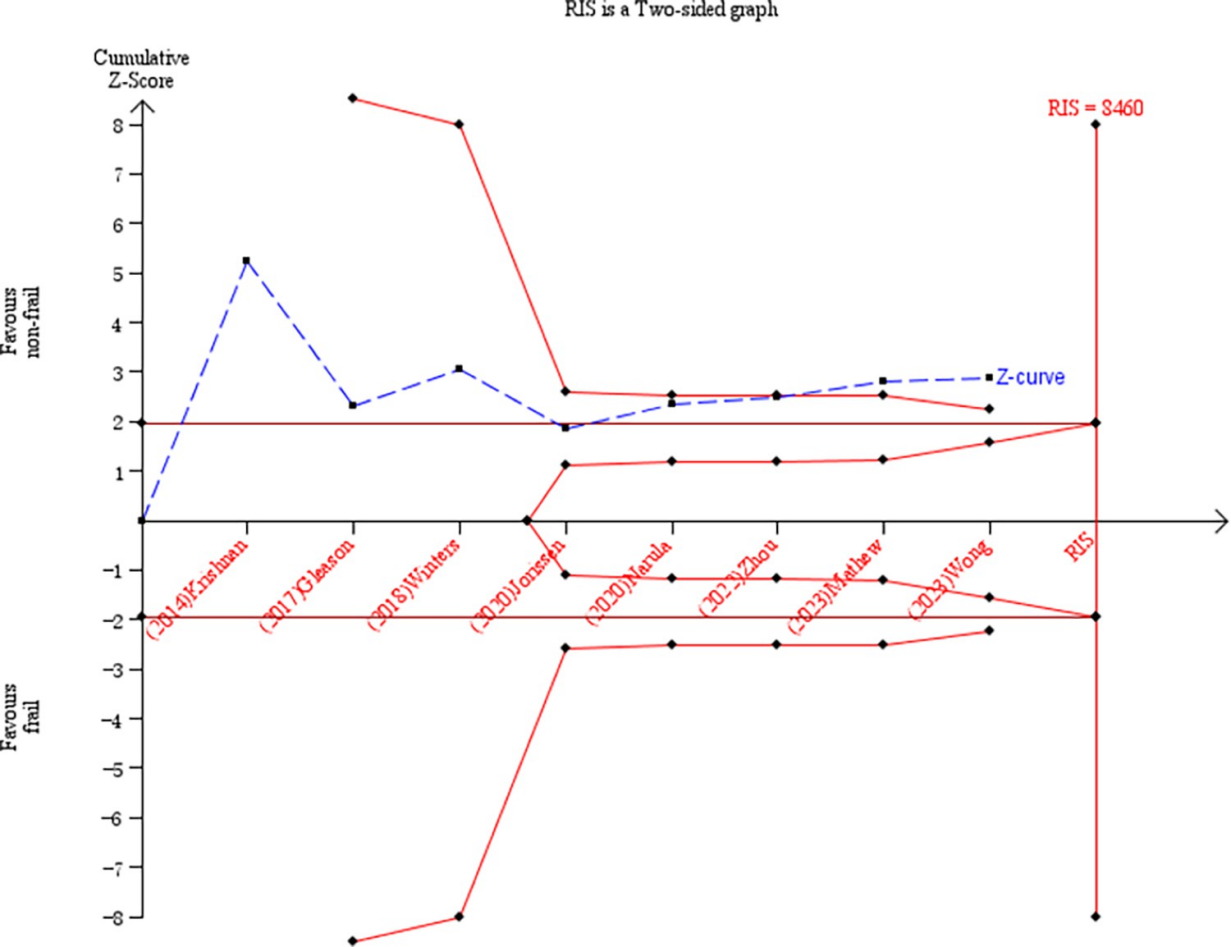
TSA of 30-day mortality predicted by frailty.

Of seven studies reporting the relationship between frailty and 30-day mortality [34–37, 43–45], the pooled results demonstrated that it was 200% higher in frail than nonfrail people (RR = 2.13, 95% CI: 1.22–3.70, *I*^2^ = 52%, *P* = 0.007; Fig 4). In the TSA, we defined the RRR and incidence in the frail group as 53.0% and 9.4%, respectively. As shown in Fig 5, the cumulative Z-curve crossed the trial sequential monitoring boundary and conventional benefit boundary. Although the current sample size (7,015) remained far below the RIS (9,192), it is noted that the current evidence is sufficient (Fig 5).

**Fig 6:**
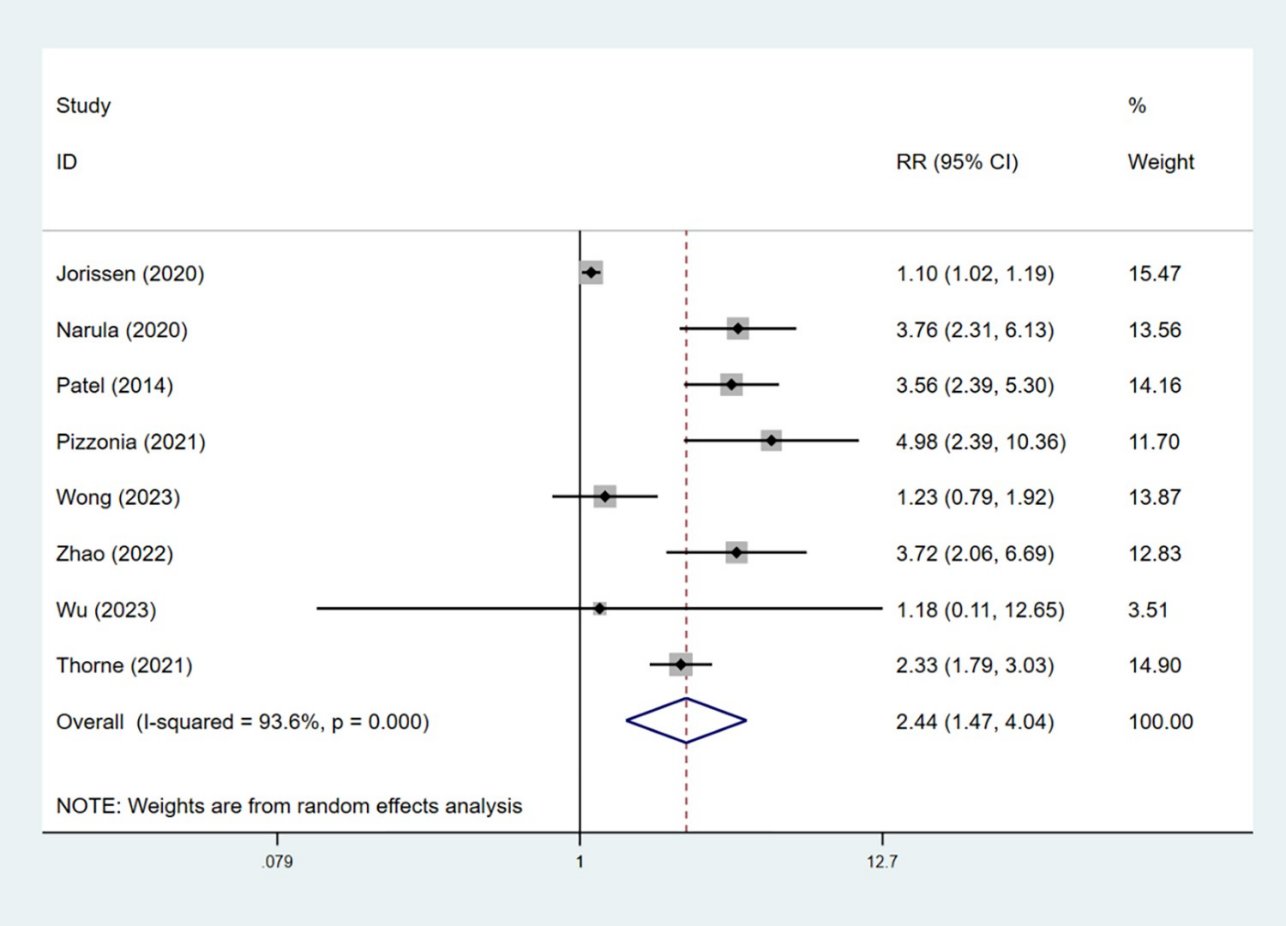
Forest plot of the RRs for the association between frailty and 1-year mortality.

**Fig 7:**
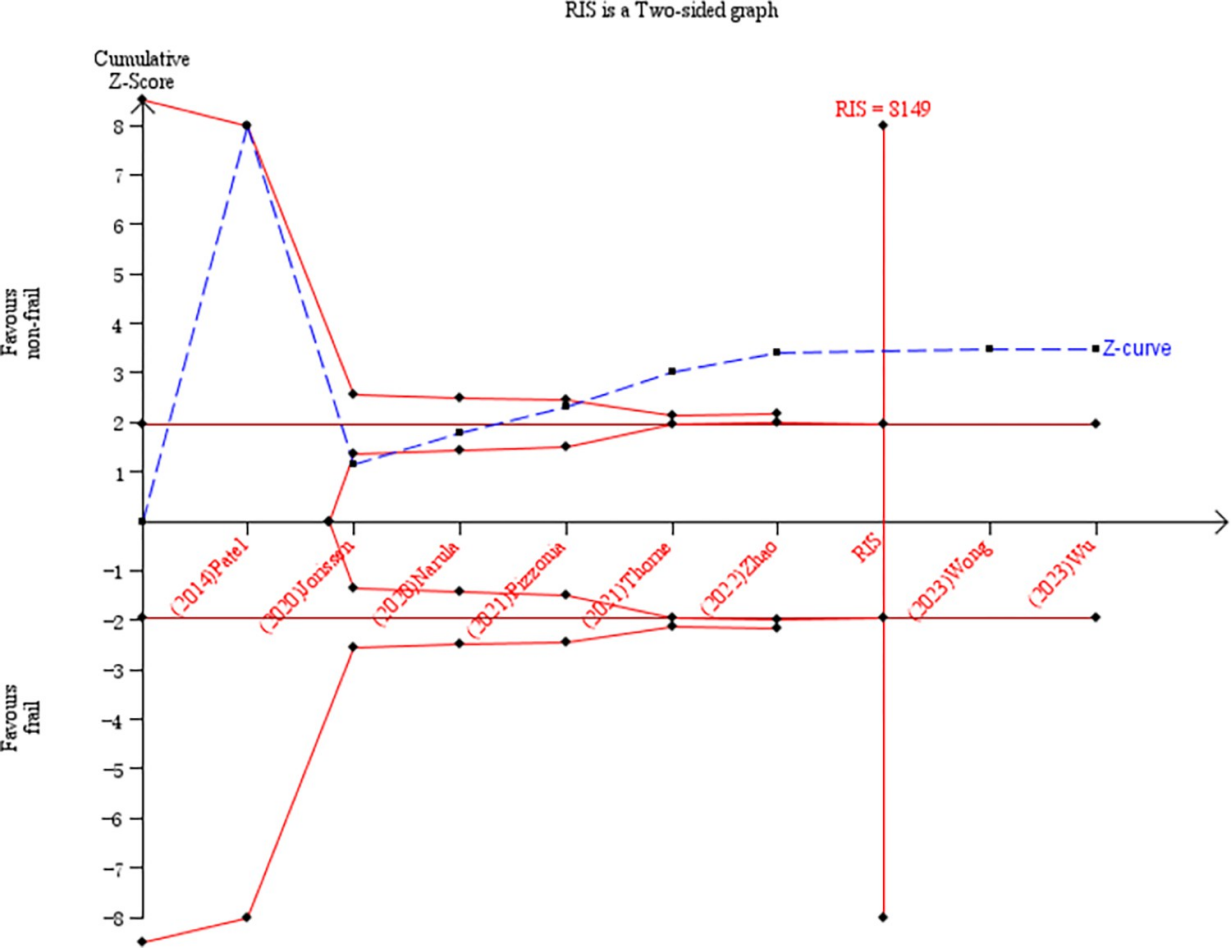
TSA of 1-year mortality predicted by frailty.

Eight studies were included to explore the association between frailty and 1-year mortality in HF patients [13, 30, 36, 37, 39, 41, 45, 46]. We noted that frailty in HF patients was associated with a significantly increased risk of 1-year mortality (RR = 2.44, 95% CI: 1.47–4.04, *I*^2^ = 94%, *P* = 0.001; Fig 6). The incidence of 1-year mortality was 31.6% and 22.7% in the frail and nonfrail groups, respectively. Keeping the risk of type I and type II errors at 5% and 20%, respectively, in the TSA, the current sample size (8,884) was greater than the RIS of 8,149. The cumulative Z-curve crossed the trial sequential monitoring boundary and conventional benefit boundary. Although the TSA diagram indicated some controversy among the selected studies, the current data are sufficient and demonstrated a statistically significant difference in 1-year mortality between the frail and nonfrail groups (Fig 7).

Meta-regression analysis and subgroup analysis of the following potential confounders were performed to examine the sources of heterogeneity: geographic region, study design, and frailty assessment tool. In terms of 30-day mortality and 1-year mortality, the results of meta-regression were not statistically significant for geographic region (*P* = 0.798, *P* = 0.319), study design (*P* = 0.637, *P* = 0.489), or frailty assessment tool (*P* = 0.405, *P* = 0.475), which thus might not be potential causes of heterogeneity. Overall, all subgroups showed increased 30-day mortality and 1-year mortality in frail participants when compared to nonfrail participants (S1 and S2 Figs). Further sensitivity analysis was performed to investigate whether any individual study exerted undue influence on the overall effect sizes. By omitting each study sequentially, the sensitivity analysis showed that frailty was still significantly correlated with the increase in 30-day mortality (S3A Fig) and 1-year mortality (S3B Fig), indicating that our results were stable and robust. Marginal evidence of publication bias was found for the association of frailty with 30-day mortality (*P* = 0.013) and 1-year mortality (*P* = 0.018) by Egger’s test.

### Postoperative complications predicted by frailty

Eight studies provided data for analyzing the influence of frailty on any postoperative complication [31–34, 40, 43, 44, 46]. A significantly increased risk of any postoperative complication was found in the frail group (RR = 1.76, 95% CI: 1.38–2.23, *I*^2^ = 53%, *P*<0.001). Several prespecified complications were also analyzed, including delirium, pneumonia, cardiac complications, deep venous thromboembolism/pulmonary embolism (DVT/PE), acute kidney injury (AKI), urinary tract infection (UTI), and surgical site infection (SSI). The forest plots and TSA diagrams are shown in the supplementary material.

Eleven studies [13, 31, 32, 34, 38, 42–46, 48] reported the association between frailty and delirium, and the pooled data showed that the combined RR of delirium was 3.34 times higher in frail patients (RR = 3.34, 95% CI: 1.41–7.96, *I*^2^ = 96%, *P* = 0.006; S4A Fig).

Raw data from thirteen studies [13, 16, 31, 32, 34, 40, 42–48] showed that frail patients had a 231% increased risk of pneumonia (RR = 2.31, 95% CI: 1.74–3.08, *I*^2^ = 55%, *P*<0.05; S4B Fig). Meta-regression analysis showed that the frailty assessment tool was correlated (*P* = 0.008) with the effect size of pneumonia.

Seven articles investigated the influence of frailty on cardiac complications, including myocardial infarction, new congestive heart failure, new arrhythmia, and heart block [31, 32, 34, 43–46]. The pooled results demonstrated that frail older individuals were more likely to experience cardiac complications (RR = 1.95, 95% CI: 1.10–3.44, *I*^2^ = 0%, *P* = 0.02; S4C Fig). Sensitivity analysis by omitting Kistler’s study, with a small sample size, showed an important change in the effect size (RR = 1.77, 95% CI: 0.97–3.23); however, the overall pooled RR of the combined studies was 1.95 (95% CI: 1.10–3.45). Publication bias was detected by Egger’s test (*P* = 0.014).

Eight studies [16, 32, 34, 43, 45–48] were included to examine the predictive value of frailty for DVT/PE. The risk of DVT/PE was significantly increased by 1.45-fold in frail older individuals (RR = 1.45, 95% CI: 0.82–2.58, *I*^2^ = 15%, *P* = 0.20; S4D Fig).

Crude data from seven studies [31, 32, 34, 43–46] indicated that frail patients had a 183% increased risk of AKI (RR = 1.83, 95% CI: 0.68–4.97, *I*^2^ = 55%, *P* = 0.23; S4E Fig). The *P* value of Egger’s test was 0.04, which suggested publication bias.

Six studies noted a relationship between frailty and UTI [16, 32, 43, 45–47]. The pooled results showed that frail older individuals were more likely to experience a UTI (RR = 3.25, 95% CI: 1.32–8.05, *I*^2^ = 76%, *P* = 0.01; S4F Fig). A sensitivity analysis showed that removing the study by Wong *et al*. (RR = 2.035, 95% CI: 1.06–3.87) resulted in an undue influence on the pooled results of the combined studies (RR = 3.25, 95% CI: 1.32–8.05).

Three studies recorded the incidence of SSI in frail subjects [32, 43, 45]. Our meta-analysis showed that it was more than 5 times higher than nonfrail patients (RR = 5.64, 95% CI: 3.85–8.27, *I*^2^ = 4%, *P*<0.05; S4G Fig).

According to TSA, given that the Z-curve did not cross the traditional threshold, monitoring boundary, or RIS boundary, more high-quality research is needed to further examine the significant correlation between frailty and delirium, pneumonia, cardiac complications, DVT/PE, AKI, and UTI (S5 Fig).

## Discussion

The present systematic review and meta-analysis with TSA was designed to examine the association between frailty and mortality as well as selected postoperative complications. Based on the meta-analysis of 21 studies of high methodological quality, we found that frailty was still a reliable factor for predicting postoperative complications such as delirium, pneumonia, cardiac complications, UTI, and SSI. There was an unclear relationship between frailty and DVT/PE and AKI.

The majority of frailty assessment instruments included in this meta-analysis were derived from the FI and CFS. The same tools were also identified in a scoping review that focused on frailty instruments used in hospitalized orthopedic populations aged over 65 [49]. Nonetheless, prevalence may vary according to the frailty assessment tool used. Based on the same study participants aged over 65 years, Kehler *et al*. [50] reported that the prevalence of frailty was 7.8% and 20.2% with the Fried and Accumulation of Deficits Model. The difference in prevalence may be explained by the various frailty models. Additionally, not all included studies reported the reliability and validity of tools. The FS is reportedly an optimal screening tool for predicting the risk of decline in health but does not mention orthopedic participants [34]. We must admit that interpretation of frailty may be influenced by orthopedic populations’ own muscle loss or weakness or postural imbalance. We noted that the Hip-MFS was amended for HF, and its intermediate quality was reported by Choi *et al*. [33]. However, there are differences in frailty measurements. The VMS was applied at or within 24 hours after admission, while RFS quantification was performed on postoperative day 1 [16]. In this meta-analysis, the degree of frailty was self-reported by most frailty instruments, and self-reported results may be underestimated by older patients. A Spanish Frail-VIG index containing self-reported or healthcare workers’ evaluation data was reported that had strong predictive impact on mortality, even patients’ situational diagnosis of life path, providing an approach of precision medicine to make personalized decisions [51]. Egglestone *et al*. [52] found that Chin-on-Chest in Neck of Femur Fracture, a simple radiographic sign, can be a predictive marker of frailty and increased mortality. Future frailty assessment tools based on multimodal evaluations may become a reality. Overall, while the available evidence supports the feasibility of using frailty assessment tools in older individuals undergoing HF surgery, further investigation into which frailty measures and which time point to measure frailty are appropriate is needed.

In this study, frailty was found to significantly affect postoperative survival among geriatric HF patients. Recent systematic reviews have compared long-term mortality and all-cause mortality, and few studies have focused on short-term mortality [1, 17]. Although 3 included studies [16, 34, 45] in our meta-analysis showed no significance between frailty and inpatient mortality, 30-day mortality, and 1-year mortality, the overall pooled results revealed an obvious trend that frailty increased the risk of mortality. This finding is consistent with that reported by Ma, who found that thirteen out of 18 articles demonstrated frailty to be significantly associated with higher inpatient mortality, 30-day mortality, and 1-year mortality [53]. Notably, the findings suggested that we should raise awareness of frailty screening and inpatient care. With much earlier screening and intervention for frailty, the risk of short-term mortality could be reduced, as well as the risk of long-term mortality and incidence of any adverse event outside of the hospital. Another important finding is that frail patients had a higher risk of 1-year mortality than 30-day mortality and inpatient mortality. Frailty is a dynamic state in which physiological, physical and social functions change over time and are mostly associated with worsening health rather than improving health [54]. Additionally, a previous study has shown that the CFS may be a reliable tool for predicting short-term mortality in emergency patients [55]. Older participants in this study were almost all enrolled from the trauma center. Subgroup analysis of the FS/CFS also revealed a higher risk of 30-day mortality and 1-year mortality. Further analysis of the diagnostic accuracy of frailty assessment tools is needed in the HF population. Although we found nothing to explain the sources of heterogeneity by meta-regression and subgroup analyses, the findings of the subgroup analysis also indicated that frailty significantly predicted mortality in prospective studies. More high-quality prospective studies are needed to confirm the validity of frailty in predicting increased mortality. This may provide in-depth information and details for improving the safety of older adults who have undergone HF surgery in the short term.

Among the 21 included studies in the current study, 15 studies reported at least one postoperative complication, including delirium, pneumonia, cardiac complications, DVT/PE, AKI, UTI, or SSI, which is consistent with the results of studies in the relevant domains [1]. In contrast to other systematic reviews, our meta-analysis expands the understanding of the relationship between frailty and various types of complications.

Delirium is the most common postoperative complication among aged patients after HF surgery, with a prevalence ranging from 4%-53.3% [56]. As shown in the present study, frailty is strongly associated with postoperative delirium, which also negatively affects functional and cognitive outcomes after hospital discharge [57]. Several known theories have been established, which consider neurotransmitter imbalance, inflammation, and electrolyte or metabolic derangement to be factors contributing to delirium [58]. In line with our included studies [56], older patients (per year increase) were more likely to experience powerful and fatal inflammatory responses, leading to a worse delirious state. Established studies on the prevention of delirium were performed from the perspective of correcting the precipitating factors of delirium. In terms of advanced age, a significant risk factor, no strategies can reverse this variable. We propose that there is a close relationship between frailty and delirium, and what we can do to protect this vulnerable population is to focus on their frailty status and explore more humanistic care management strategies to promote healthy aging.

In line with results reported by Gao *et al*. [59], our pooled results revealed a higher prevalence of delirium and pneumonia. Among the meta-regression analyses, frailty measures significantly affected the pooled estimates in terms of predicting pneumonia (*P* = 0.008). Further regression analysis was performed with the frailty assessment tool as the dummy variable, nevertheless, no significant difference was found. Moreover, Park has shown that the Frailty Index combined with the Pneumonia Severity Index significantly improved the prediction [60], indicating that the clinical characteristics of disease-related complications should also be considered when exploring new frailty risk stratification tools in specific populations.

The increased systemic inflammatory response after trauma [61] may lead to trauma-induced secondary cardiac injuries and events, including [62] acute coronary syndrome, atrial fibrillation, and ventricular arrhythmia, which is consistent with our results. Studies have shown that advanced age (>75 years), elevated systemic levels of troponin I, brain natriuretic peptide, and heart fatty acid binding protein, and reduced ejection fraction may be independent risk factors for predicting the occurrence of major adverse cardiac events following HF [62]. Furthermore, given that the pathophysiological mechanism of frailty is still unclear, perhaps developing a frailty assessment tool involving the abovementioned specific laboratory values would increase the value of frailty in predicting adverse cardiac outcomes among HF patients. Although older frail patients seem likely to experience cardiac complications after HF surgery (RR = 1.95, 95% CI: 1.10–3.4), the result was not reliable. After omission of Kistler’s study, the pooled results were robust and stable. Kistler *et al*. conducted interviews with participants to recall their prefracture status and then obtained frailty scores, and this process may have involved bias, impacting the overall findings. Additionally, all participants in Kistler’s study were enrolled from the Geriatric Fracture Center, where a multidisciplinary team including orthopedic surgeons and geriatricians act as consultants, and the original results showed no significant difference between frailty and any complication. A review has shown that compared to the geriatric model of care, a trend toward a reduction in complications was presented by an integrated orthogeriatric model [63], which may provide more details about the reason why Kistler’s study strongly affected the pooled estimates.

Pooled analysis demonstrated no significant effect of frailty on DVT/PE. The cause of the poor association may derive from insufficient longitudinal follow-up data. The types of HF in this study included intracapsular femoral neck fractures, intertrochanteric fractures, subtrochanteric fractures, and proximal femoral fractures. A previous study reported that DVT/PE is commonly caused by severe vascular injury correlated with a type of high-energy injury in complex subtrochanteric fractures [64]. Notably, due to prolonged nonweight bearing, extensive tissue damage, and a hypercoagulable state [65], older HF patients are still at high risk of developing DVT/PE, which may lead to postthrombotic syndrome and a high risk of disability, eventually leading to increased mortality [64].

Older frail patients are likely to experience a UTI after HF surgery because of their immobility and frequently undergo catheterization [66]. Suen *et al*. [67] carried out a systematic review and found that HF patients with a perioperative UTI were more likely to experience a 2.4-fold increased risk of SSI. The cases of participants in this meta-analysis were complex, considering the presence of multiple comorbidities, such as hypertension, diabetes, and dementia. Moreover, as shown in Bohl’s study, UTI, pneumonia, and SSI are important contributors to sepsis [68]; thus, healthcare workers should take care to monitor the state of consciousness of older frail HF patients.

## Strengths and limitations

To our knowledge, this is the first systematic review and meta-analysis with TSA to investigate the link between frailty and postoperative adverse outcomes among older HF patients. The strength of the current study is its strict inclusion and exclusion criteria. We focused on those over 60 years old who were surgically treated for HF. Studies that mixed younger and older patients undergoing conservative treatment were excluded. Moreover, some studies that enrolled only patients undergoing hip arthroplasty were excluded. In addition, sensitivity analyses showed that most results were robust and reliable. TSA demonstrated that the number of included studies was adequate for inpatient mortality, 1-year mortality, and pneumonia.

Some limitations should be noted. First, we only included articles published in English and Chinese. Second, 13 out of twenty-one included studies were retrospective and inevitably involved interviewer and recall bias. Third, studies used various frailty definitions as well as assessment scales and cutoffs, which may have introduced heterogeneity. Additionally, several included studies did not report adjusted estimates; therefore, the pooled estimates may be biased due to various confounding factors. Finally, due to the limited sample size, we did not conduct a sensitivity analysis of inpatient mortality or SSI. The type of HF was not used to perform subgroup analysis owing to insufficient data. Although we analyzed subgroups based on geographic region, study design, and frailty assessment tool, sources of heterogeneity were not observed. Therefore, the findings should be interpreted with caution.

## Conclusion

This meta-analysis found that frailty was a powerful predictor of inpatient mortality, 30-day mortality, 1-year mortality, and delirium, pneumonia, cardiac complications, UTI, and SSI, but not DVT/PE or AKI, as postoperative complications in older patients after HF surgery. It is obvious that frailty holds promise to serve as a triage tool for helping healthcare workers determine optimal treatment regimens among conservative and surgical treatments. Frailty assessment tools tailored to this specific HF population are pivotal. In addition, early accurate detection, prevention, and treatment of frailty should be added to orthogeriatric management models, which should be emphasized in the care of older frail patients with HF, thus promoting healthy aging.

## Supporting information

S1 Table PRISMA 2020 checklist. (PDF)

S2 Table Search strategy for PubMed. (PDF)

S3 Table Characteristics of included studies. (PDF)

S4 Table Results of the Newcastle-Ottawa Scale quality assessment of cohort studies. (PDF)

S1 Fig Subgroup analyses of the studies reporting 30-day mortality based on geographic region, study design, and frailty assessment tool. (PDF)

S2 Fig Subgroup analyses of the studies reporting 1-year mortality based on geographic region, study design, and frailty assessment tool. (PDF)

S3 Fig Sensitivity analysis of studies reporting 30-day mortality/1-year mortality. (A) Sensitivity analysis of studies reporting 30-day mortality. (B) Sensitivity analysis of studies reporting 1-year mortality.
(PDF)

S4 Fig The funnel plot of the risk ratios of the association of frailty and postoperative complications. (A) Delirium. (B) Pneumonia. (C) Cardiac complications. (D) Deep venous thrombosis or pulmonary embolism. (E) Acute kidney injury. (F) Urinary tract infection. (G) Surgical site infection.
(PDF)

S5 Fig Trial sequential analysis for postoperative complications predicted by frailty. (A) Delirium. (B) Pneumonia. (C) Cardiac complications. (D) Deep venous thrombosis or pulmonary embolism. (E) Acute kidney injury. (F) Urinary tract infection.
(PDF)
