# Supplementary material for: Association of frailty with adverse outcomes in surgically treated geriatric patients with hip fracture: A meta-analysis and trial sequential analysis
Source: PLoS One. 2024 Jun 21;19(6):e0305706. doi: 10.1371/journal.pone.0305706 (PMC11192356; doi:10.1371/journal.pone.0305706)
Supplement: S2 Table — (PDF) [file pone.0305706.s002.pdf]

S2 Table. Search strategy for PubMed.

Search strategy for PubMed	
#1	"aged"[MeSH Terms] OR "geriatric"[Title/Abstract] OR "elderly"[Title/Abstract] OR "older adult*"[Title/Abstract] OR "older people"[Title/Abstract] OR "elder*"[Title/Abstract] OR "aged"[Title/Abstract] OR "aging"[Title/Abstract] OR "old*"[Title/Abstract] OR "old people"[Title/Abstract] OR "advanced age"[Title/Abstract] OR "elderly patients"[Title/Abstract] OR "old"[Title/Abstract] OR "older adults"[Title/Abstract] OR "older"[Title/Abstract] OR "old age"[Title/Abstract]
#2	"hip fractures"[MeSH Terms] OR "hip fracture*"[Title/Abstract] OR "trochanteric fracture*"[Title/Abstract] OR "intertrochanteric fracture*"[Title/Abstract] OR "subtrochanteric fracture*"[Title/Abstract] OR "proximal femoral fracture*"[Title/Abstract] OR "femoral neck fracture*"[Title/Abstract] OR "femoral head fracture*"[Title/Abstract] OR "acetabulum fracture*"[Title/Abstract] OR "hip joint fracture*"[Title/Abstract]
#3	#1 AND #2
#4	"postoperative period"[MeSH Terms] OR "postoperative period*"[Title/Abstract] OR "postoperative"[Title/Abstract] OR "post surgical"[Title/Abstract] OR "postsurgical"[Title/Abstract] OR "surgery past"[Title/Abstract]
#5	"frailty"[MeSH Terms] OR "frail*"[Title/Abstract] OR "frailty syndrome*"[Title/Abstract] OR "weakness*"[Title/Abstract] OR "debilit*"[Title/Abstract] OR "vulnerabilit*"[Title/Abstract]
#6	#4 AND #5
#7	#3 AND #6
