# Supplementary material for: Association of frailty with adverse outcomes in surgically treated geriatric patients with hip fracture: A meta-analysis and trial sequential analysis
Source: PLoS One. 2024 Jun 21;19(6):e0305706. doi: 10.1371/journal.pone.0305706 (PMC11192356; doi:10.1371/journal.pone.0305706)
Supplement: S3 Table — (PDF) [file pone.0305706.s003.pdf]

S3 Table. Characteristics of included studies.

First author, year	Study design	Country	Setting	Period	Sample size	Age/years	Sex, Female, n(%)	Frailty assessment tool	Prevalence of frailty	Outcomes
Patel 2014	retrospective	USA	an American College of Surgeons-verified Level I trauma center	2005-2009	481	81.05±8.45（60-105)	-	modified Frailty Index-19 items (mFI-19)	41.6%	1-year mortality
Kistler 2016	prospective	USA	a university-affiliated community hospital with Level III trauma center	June 2, 2013-August 30, 2013	35	86 ±4	29(83%)	modified Fried Frailty Index（mFFI)	51%	delirium, pneumonia, cardiac complications, acute kidney injury, any complication
Kua 2016	prospective	Singapore	the Orthopaedic Surgery Department in Tan Tock Seng Hospital	January 1, 2013- June 30, 2013	82	78.2±8.5（60-100）	55(67.1%)	Reported Edmonton Frail Scale (REFS)	34.15%	delirium, pneumonia, cardiac complications, DVT/PE, acute kidney injury, urinary tract infection, surgical site infection, any complication
Choi 2017	retrospective	Korea	Seoul National University Bundang Hospital	March 2009-May 2014	481	80.4 (75.3,85.3)	343(71.3%)	Hip-Multidimensional Frailty Score（Hip-MFS)	24.30%	any complication
Gleason 2017	retrospective	USA	a level I trauma center	August 2015 -May 2016	175	82.3±7.4	131(74.9%)	FRAIL Scale (FS)	41.71%	30-day mortality, delirium, pneumonia, cardiac complications, DVT/PE, acute kidney injury, any complication
Winters 2018	prospective	Netherlands	a general hospital	November 2014-December 2015	286	83±6.6	215（75%）	Veiligheids Management System Frailty Score (VMS)	57.76%	30-day mortality
Jorissen 2020	retrospective	Australia	the Historical cohort of the Registry of Senior Australians	July 1, 2003-December 31, 2015	4771	86 (82,90)	3607（75.6%）	Frailty Index (FI)	48.33%	30-day mortality, 1-year mortality
Narula 2020	retrospective	Australia	the tertiary referral center	November 1, 2017-October 31, 2018	509	82.7±9.1	374(73.5%)	Clinical Frailty Scale (CFS)	66.4%	in-patient mortality, 30-day mortality, 1-year mortality

You 2020	prospective	China	the Orthopaedic Unit at People's Hospital of Jiangxi Province	January 1, 2017-January 1, 2019	200	73.21±6.41	104（52%）	Frailty Index (FI)	69%	pneumonia, DVT/PE, urinary tract infection
Zhu 2020	prospective	China	the First Affiliated Hospital of Anhui Medical University	November 2019-January 2020	120	78.9±6.5	55（45.83%）	FRAIL Scale (FS)	15%	delirium, pneumonia, DVT/PE
Gandossi 2021	prospective	Italy	Orthogeriatric unit at hospital	October 1, 2011-March 15, 2019	988	84.9（80.6,89.2）	738（74.7%）	Frailty Index (FI)	36.40%	delirium
Pizzonia 2021	prospective	Italy	the Orthopaedic and Trauma Unit	September 1, 2015-June 1, 2017	364	86.57±5.65（72-102）	284（76%）	modified 19-item Frailty Index (mFI-19)	72.50%	1-year mortality
Shen 2021	retrospective	China	the Department of Orthopedics	December 2010-June 2017	965	76.77±8.76（60-100)	637（66%）	modified Frail Index (mFI)	13.06%	pneumonia, any complication
Thorne 2021	prospective	UK	two non-specialist hospitals	2016-2018	1299	–	–	Clinical Frailty Scale (CFS)	63.28%	in-patient mortality, 1-year mortality
Shimizu 2022	retrospective	Japan	a hospital-based database created by the Japan Medical Data Center (JMDC)	April 2014-August 2020	36192	83.6±6.7	28772（79.5%）	Hospital Frailty Risk Score (HFRS)	31.58%	in-patient mortality, delirium, pneumonia
Zhao 2022	retrospective	China	Peking University People’s Hospital, Beijing	June 1, 2019-May 31, 2020	381	aged⩾65	269（70.6%）	Chart-derived Frailty Index (CFI)	26.77%	1-year mortality, delirium, pneumonia
Zhou 2022	retrospective	China	the Affiliated Hospital of Medical College of Ningbo University	2015-2021	150	aged⩾60	104（69.3%）	modified Frailty Index (5-mFI)	89.30%	30-day mortality, delirium, pneumonia, cardiac complications, DVT/PE, acute kidney injury, urinary tract infection, surgical site infection, any complication
Mathew 2023	retrospective	India	a single tertiary care center	April 1, 2021- April 30, 2022	109	aged⩾65	77(70.6%)	Clinical Frailty Scale (CFS)	63.30%	30-day mortality, delirium, pneumonia, cardiac complications, acute kidney injury, any complication
Sang 2023	retrospective	USA	in a state and American College of Surgeons (ACS)–	January 2018-January 2020	470	aged⩾66	319(67.9%)	Rockwood Frailty Score (RFS)	6.60%	in-patient mortality, pneumonia, DVT/PE, urinary tract infection

			verified Level 2 trauma center (TC)							
Wong 2023	retrospective	Singapore	a tertiary hospital in Singapore	January 1, 2016-June 30, 2020	1014	aged⩾60	732 (72.2%)	Hospital Frailty Risk Score (HFRS)	41.81%	30-day mortality,1-year mortality, delirium, pneumonia, cardiac complications, DVT/PE, acute kidney injury, urinary tract infection, surgical site infection
Wu 2023	retrospective	China	Guangdong Province Hospital of Chinese Medicine	December 2019-August 2021	124	82.79±7.53(65-102)	92(74.19%)	Laboratory Frailty Index (FI-lab)	62.90%	1-year mortality, delirium, pneumonia, cardiac complications, DVT/PE, , acute kidney injury, urinary tract infection, any complication
