# Supplementary material for: Association of frailty with adverse outcomes in surgically treated geriatric patients with hip fracture: A meta-analysis and trial sequential analysis
Source: PLoS One. 2024 Jun 21;19(6):e0305706. doi: 10.1371/journal.pone.0305706 (PMC11192356; doi:10.1371/journal.pone.0305706)
Supplement: S2 Fig — (PDF) [file pone.0305706.s006.pdf]

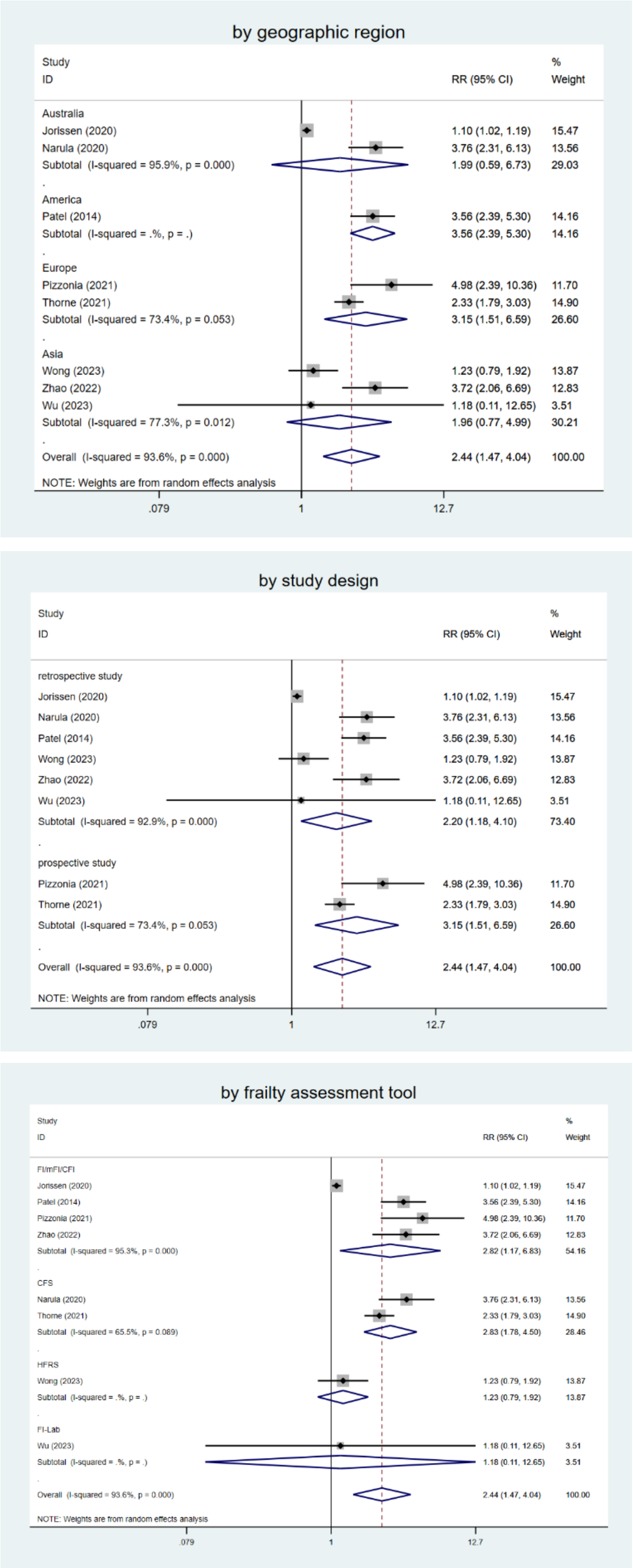

S2 Fig. Subgroup analyses of the studies reporting 1-year mortality based on geographic region, study design, and frailty assessment tool.
