# Supplementary material for: Association of frailty with adverse outcomes in surgically treated geriatric patients with hip fracture: A meta-analysis and trial sequential analysis
Source: PLoS One. 2024 Jun 21;19(6):e0305706. doi: 10.1371/journal.pone.0305706 (PMC11192356; doi:10.1371/journal.pone.0305706)
Supplement: S3 Fig — (A) Sensitivity analysis of studies reporting 30-day mortality. (B) Sensitivity analysis of studies reporting 1-year mortality. (PDF) [file pone.0305706.s007.pdf]

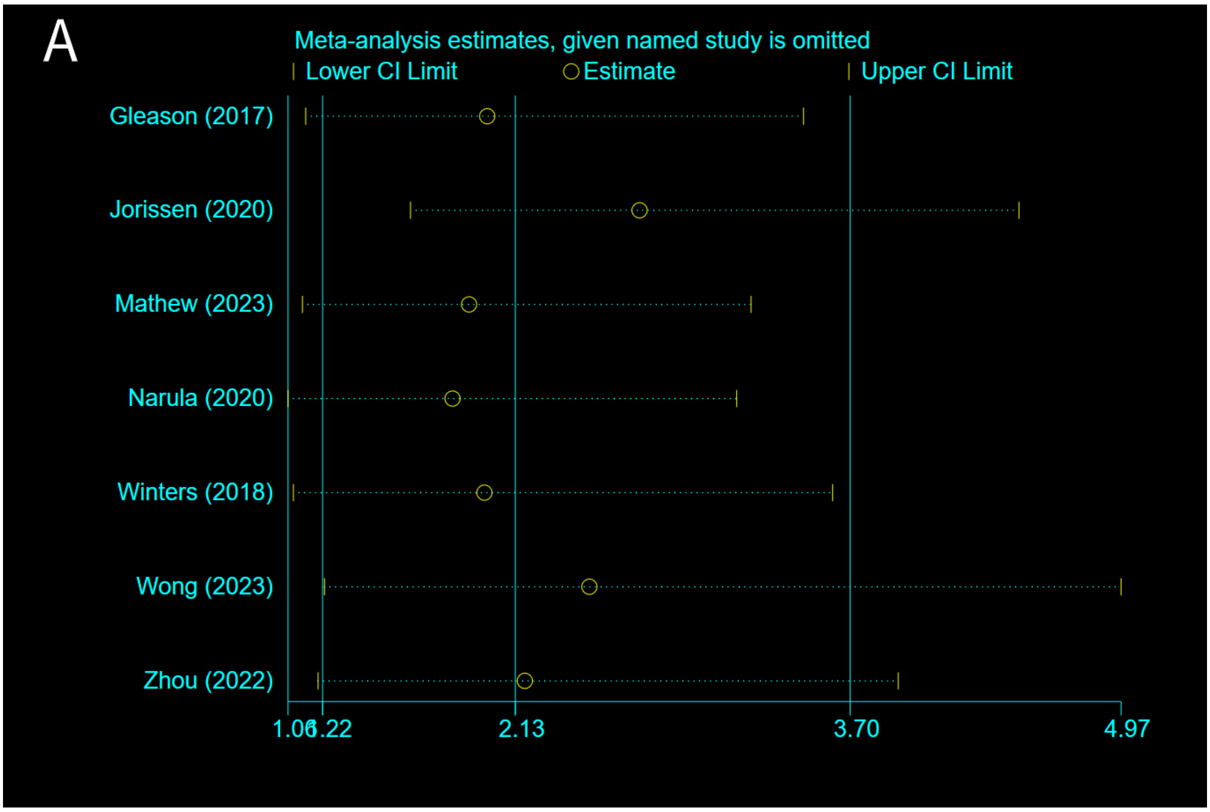

S3A Fig. Sensitivity analysis of studies reporting 30-day mortality.

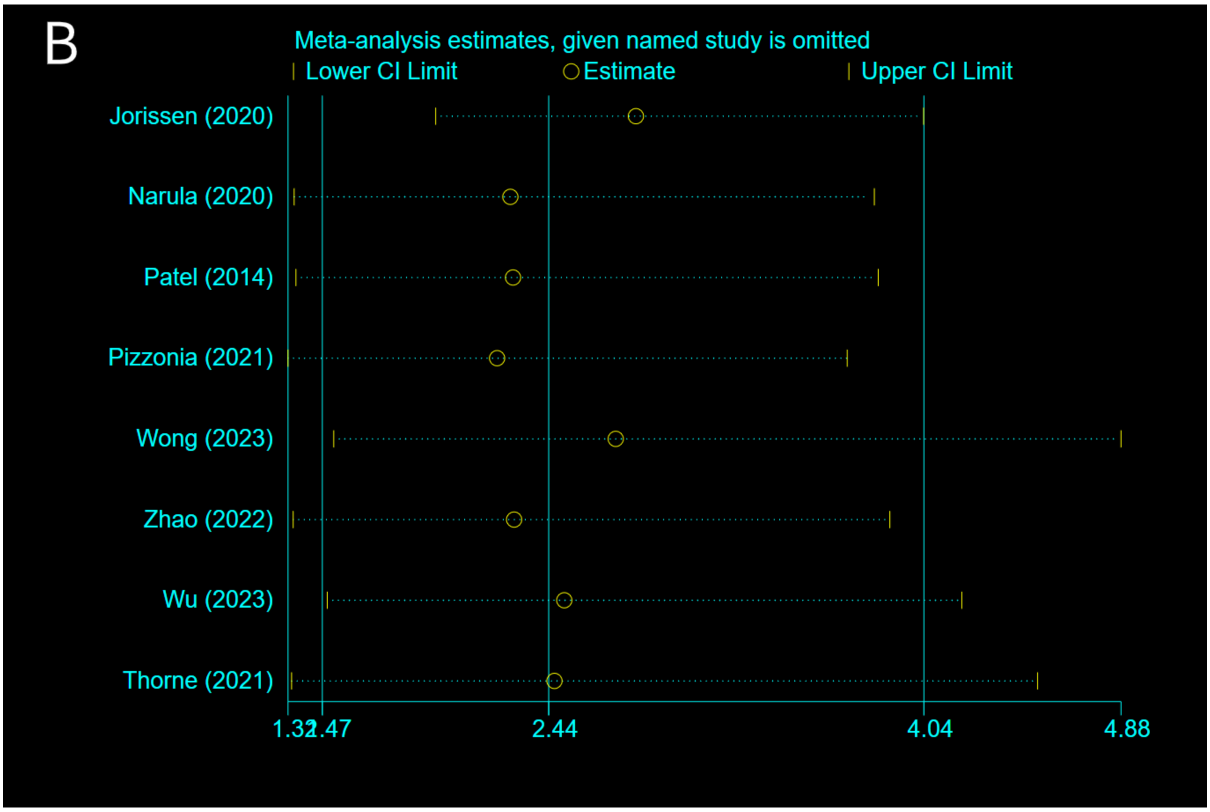

S3B Fig. Sensitivity analysis of studies reporting 1-year mortality.
