# Supplementary material for: Association of frailty with adverse outcomes in surgically treated geriatric patients with hip fracture: A meta-analysis and trial sequential analysis
Source: PLoS One. 2024 Jun 21;19(6):e0305706. doi: 10.1371/journal.pone.0305706 (PMC11192356; doi:10.1371/journal.pone.0305706)
Supplement: S4 Fig — (A) Delirium. (B) Pneumonia. (C) Cardiac complications. (D) Deep venous thrombosis or pulmonary embolism. (E) Acute kidney injury. (F) Urinary tract infection. (G) Surgical site infection. (PDF) [file pone.0305706.s008.pdf]

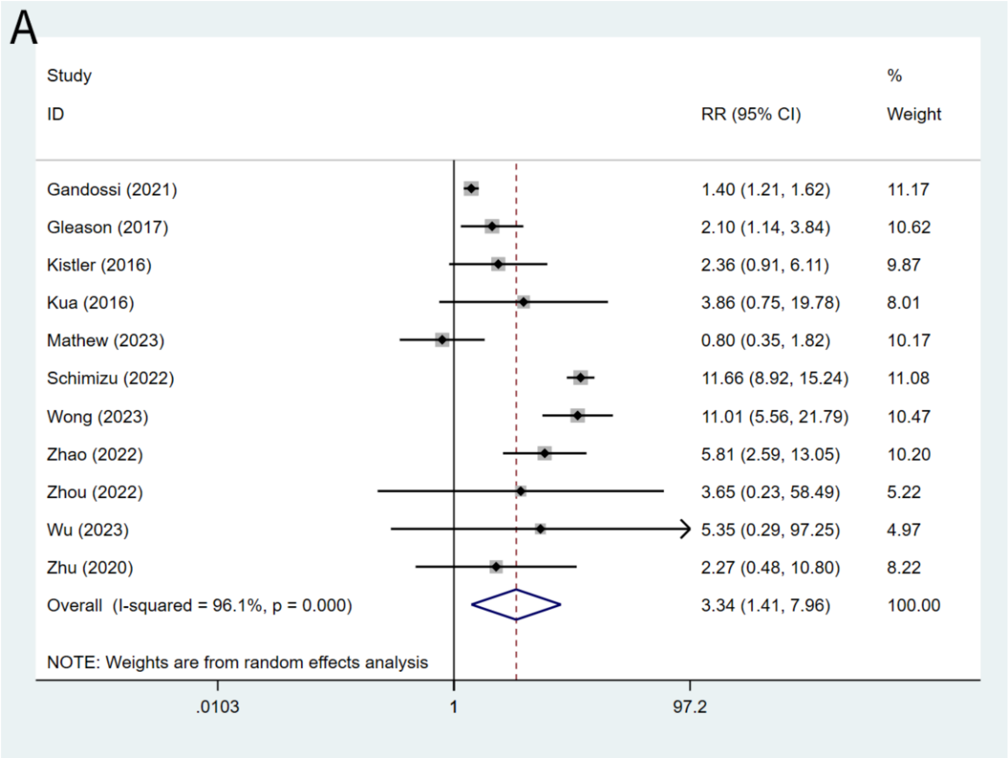

S4A Fig. Forest plot of the risk ratios for the association between frailty and delirium.

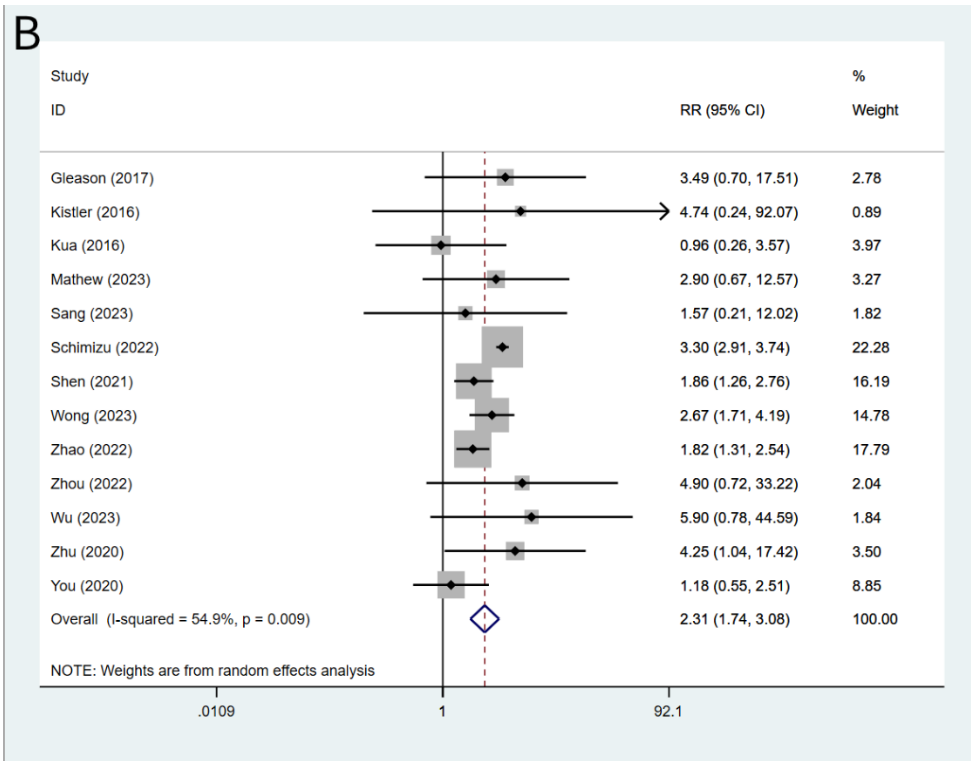

S4B Fig. Forest plot of the risk ratios for the association between frailty and pneumonia.

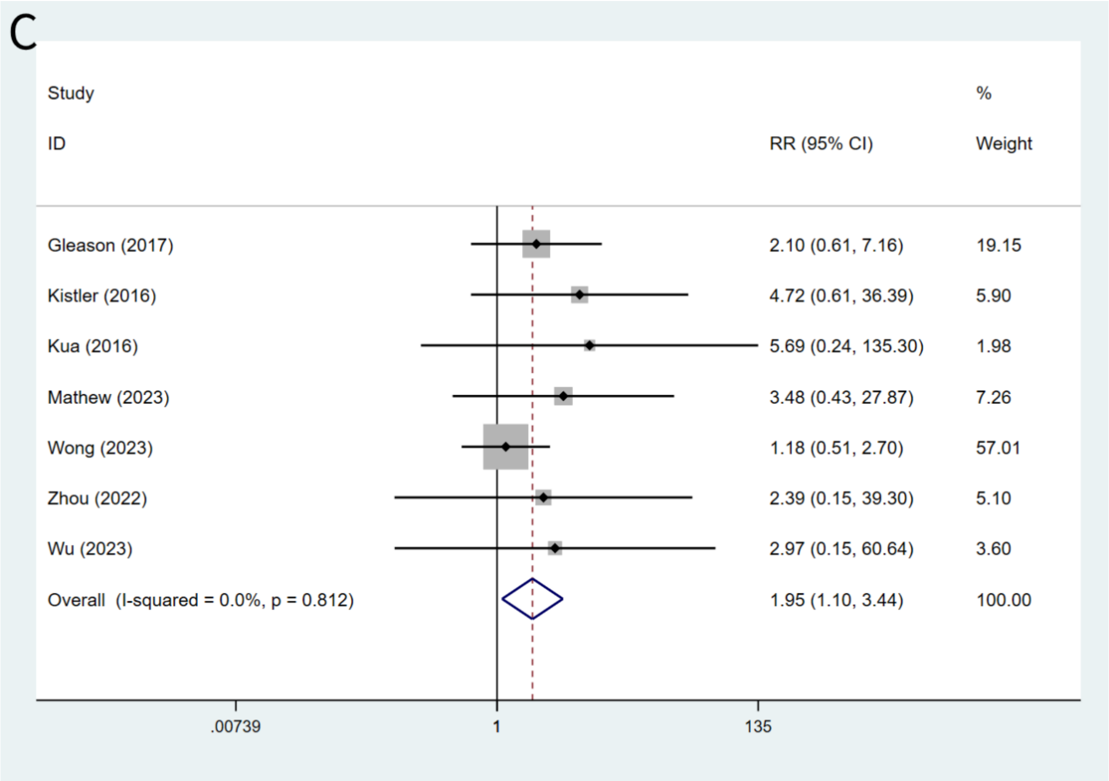

S4C Fig. Forest plot of the risk ratios for the association between frailty and cardiac complications.

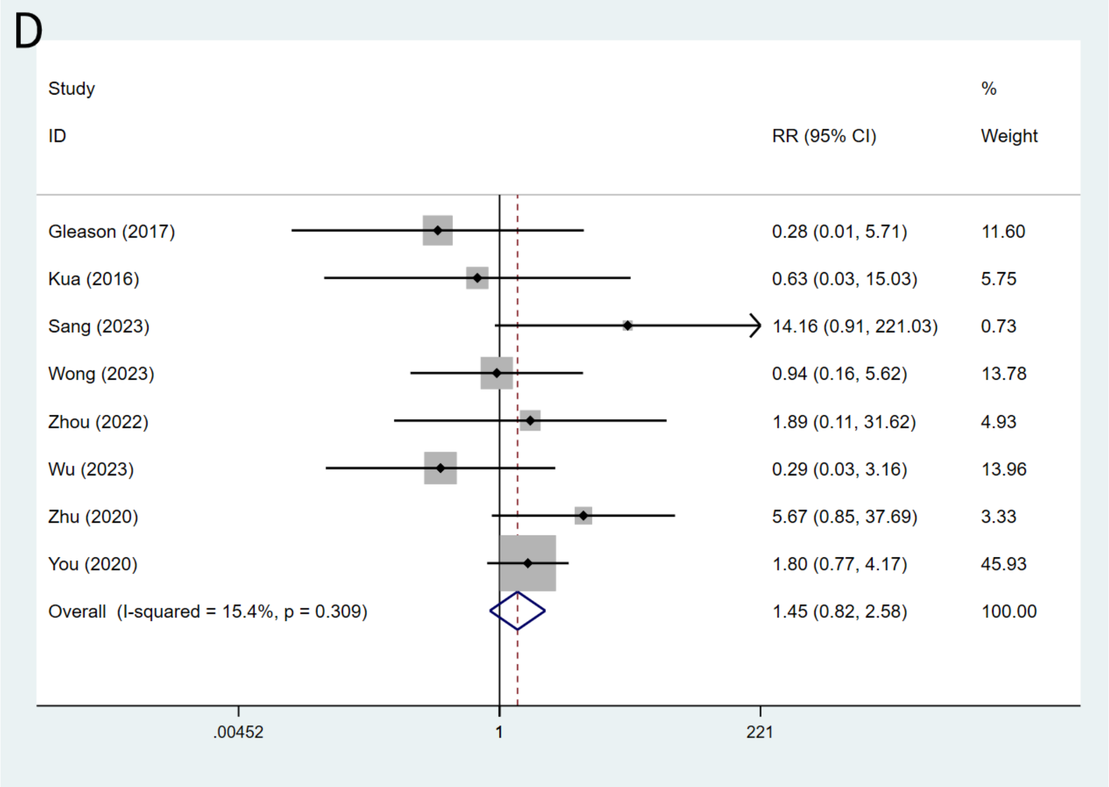

S4D Fig. Forest plot of the risk ratios for the association between frailty and deep venous thrombosis or pulmonary embolism.

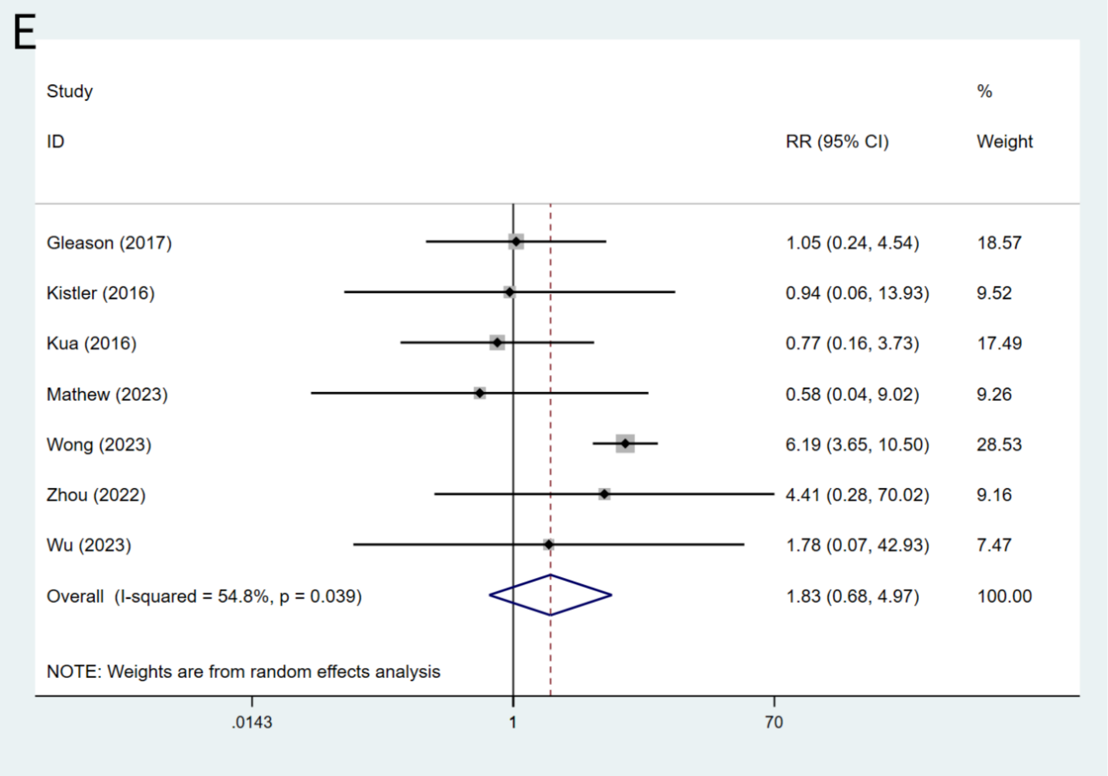

S4E Fig. Forest plot of the risk ratios for the association between frailty and acute kidney injury.

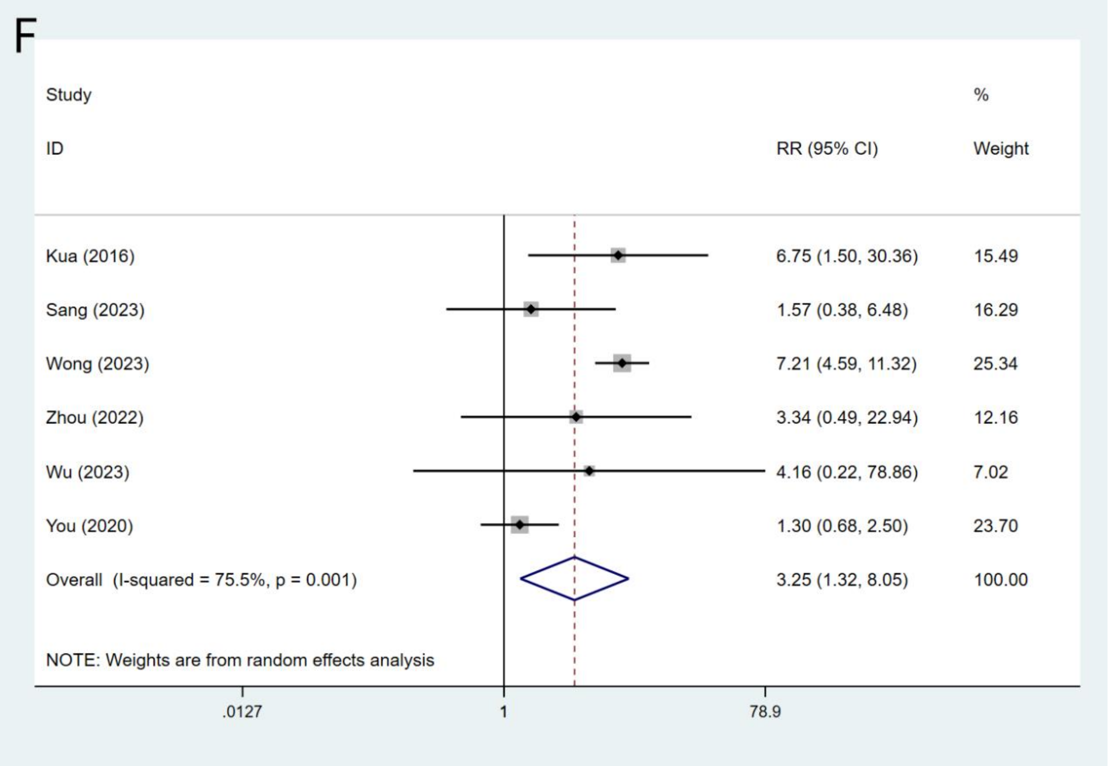

S4F Fig. Forest plot of the risk ratios for the association between frailty and urinary tract infection.

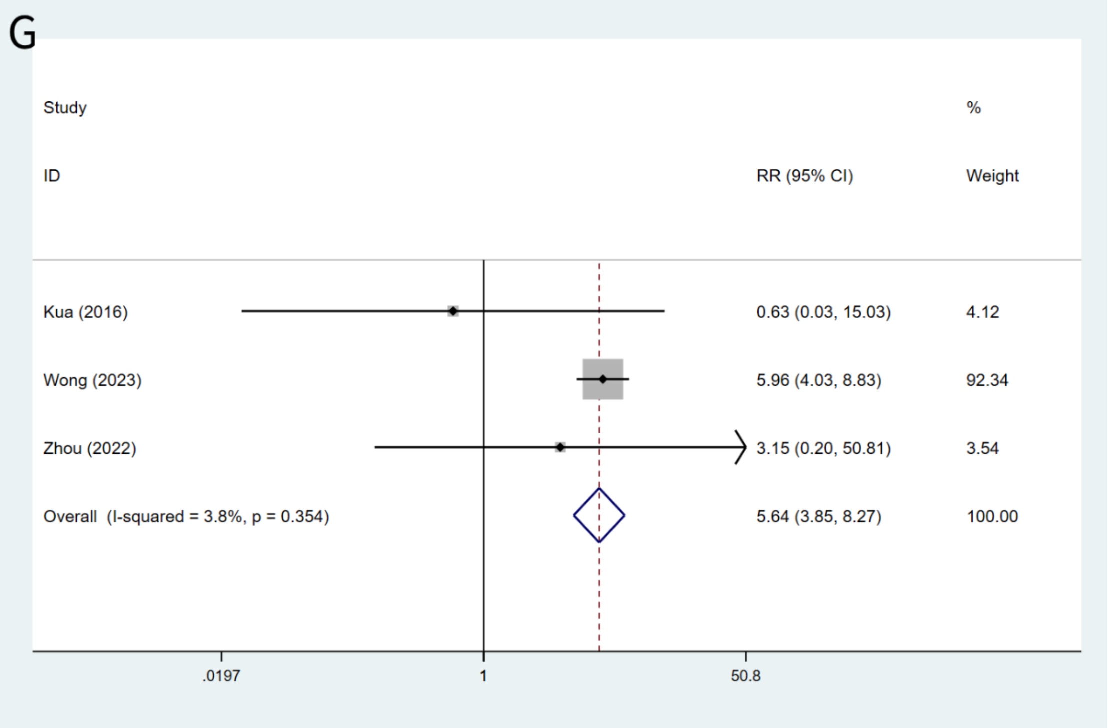

S4G Fig. Forest plot of the risk ratios for the association between frailty and surgical site infection.
