# Supplementary material for: Association of frailty with adverse outcomes in surgically treated geriatric patients with hip fracture: A meta-analysis and trial sequential analysis
Source: PLoS One. 2024 Jun 21;19(6):e0305706. doi: 10.1371/journal.pone.0305706 (PMC11192356; doi:10.1371/journal.pone.0305706)
Supplement: S5 Fig — (A) Delirium. (B) Pneumonia. (C) Cardiac complications. (D) Deep venous thrombosis or pulmonary embolism. (E) Acute kidney injury. (F) Urinary tract infection. (PDF) [file pone.0305706.s009.pdf]

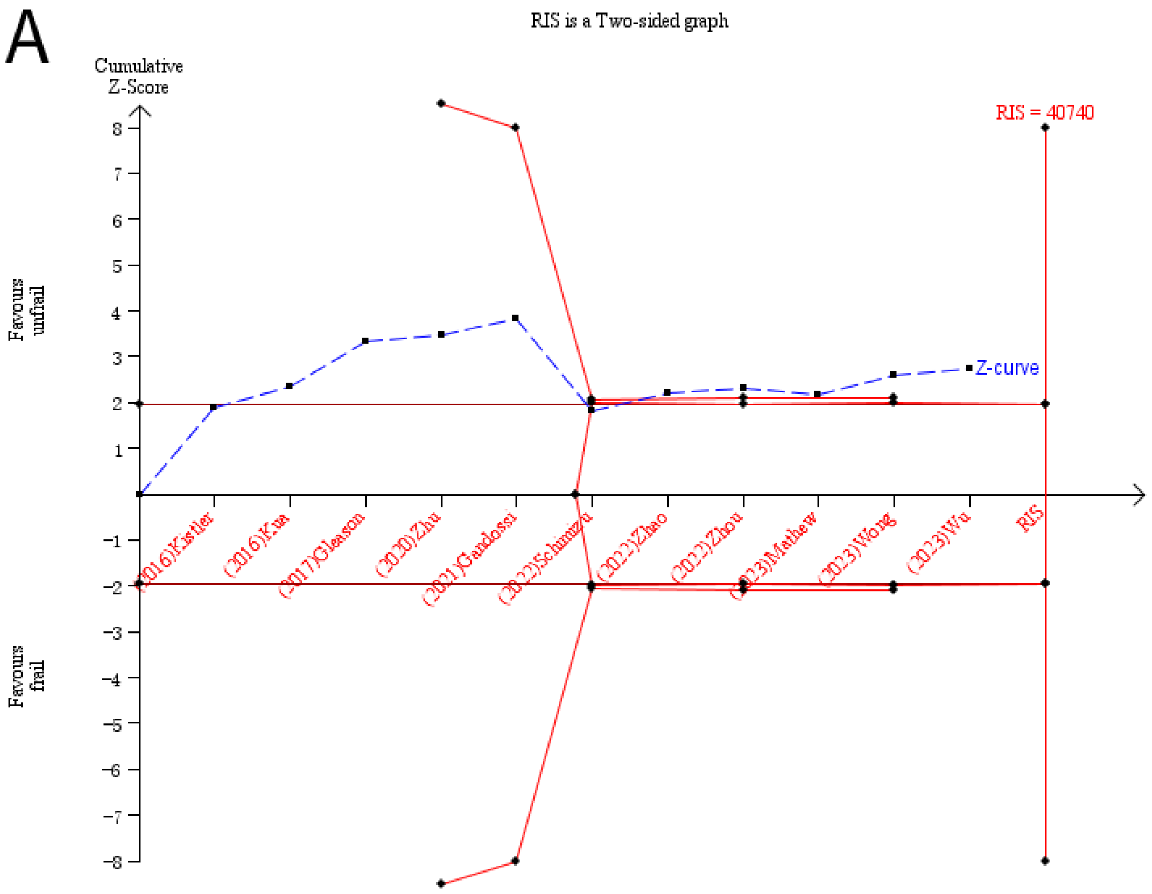

S5A Fig. Trial sequential analysis for delirium predicted by frailty. The diversity-adjusted required information size of 40740 participants was based on a type of I error of 5%, a type of II error of 20%, and a control group (frail participants) proportion of 5.3%, with a Relative Risk Reduction of 70%.

B

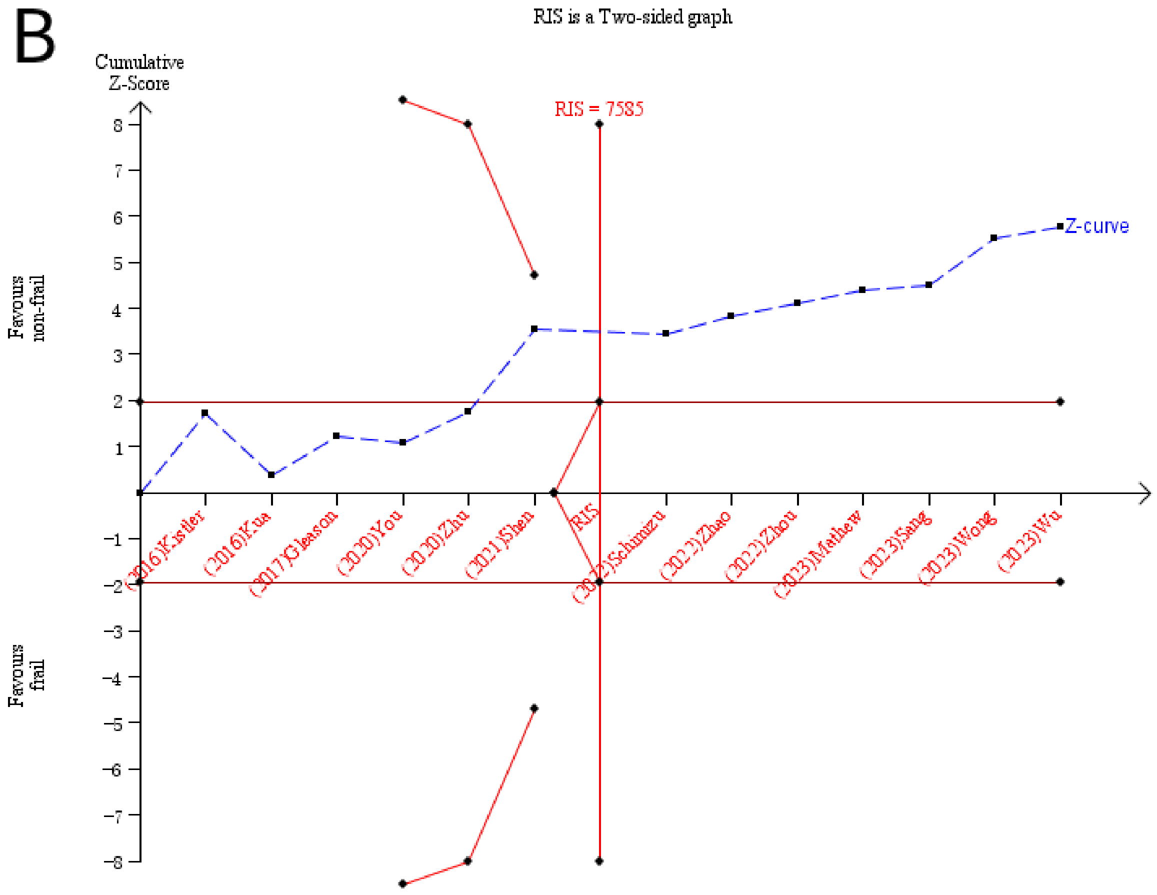

S5B Fig. Trial sequential analysis for pneumonia predicted by frailty. The diversity-adjusted required information size of 7585 participants was based on a type of I error of 5%, a type of II error of 20%, and a control group (frail participants) proportion of 6.3%, with a Relative Risk Reduction of 57%.

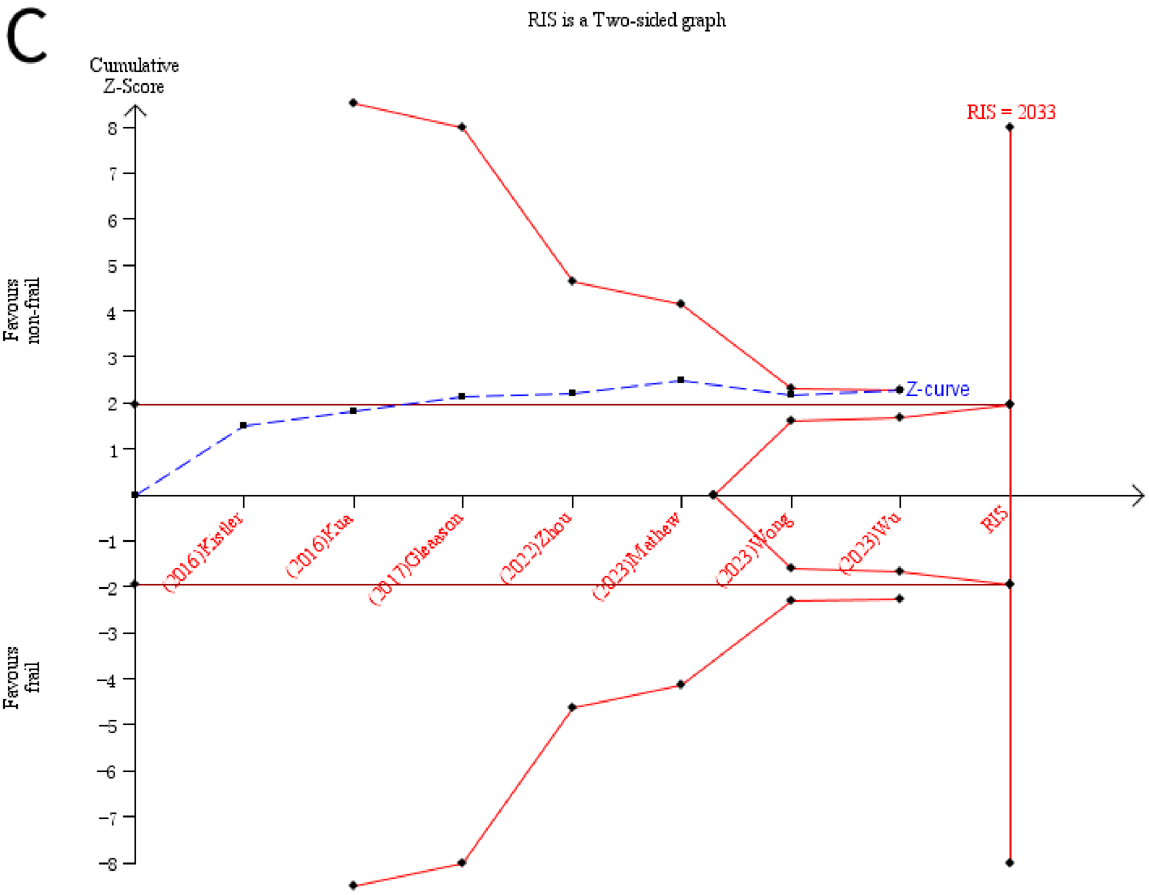

S5C Fig. Trial sequential analysis for cardiac complications predicted by frailty. The diversity-adjusted required information size of 2033 participants was based on a type of I error of 5%, a type of II error of 20%, and a control group (frail participants) proportion of 4.7%, with a Relative Risk Reduction of 49%.

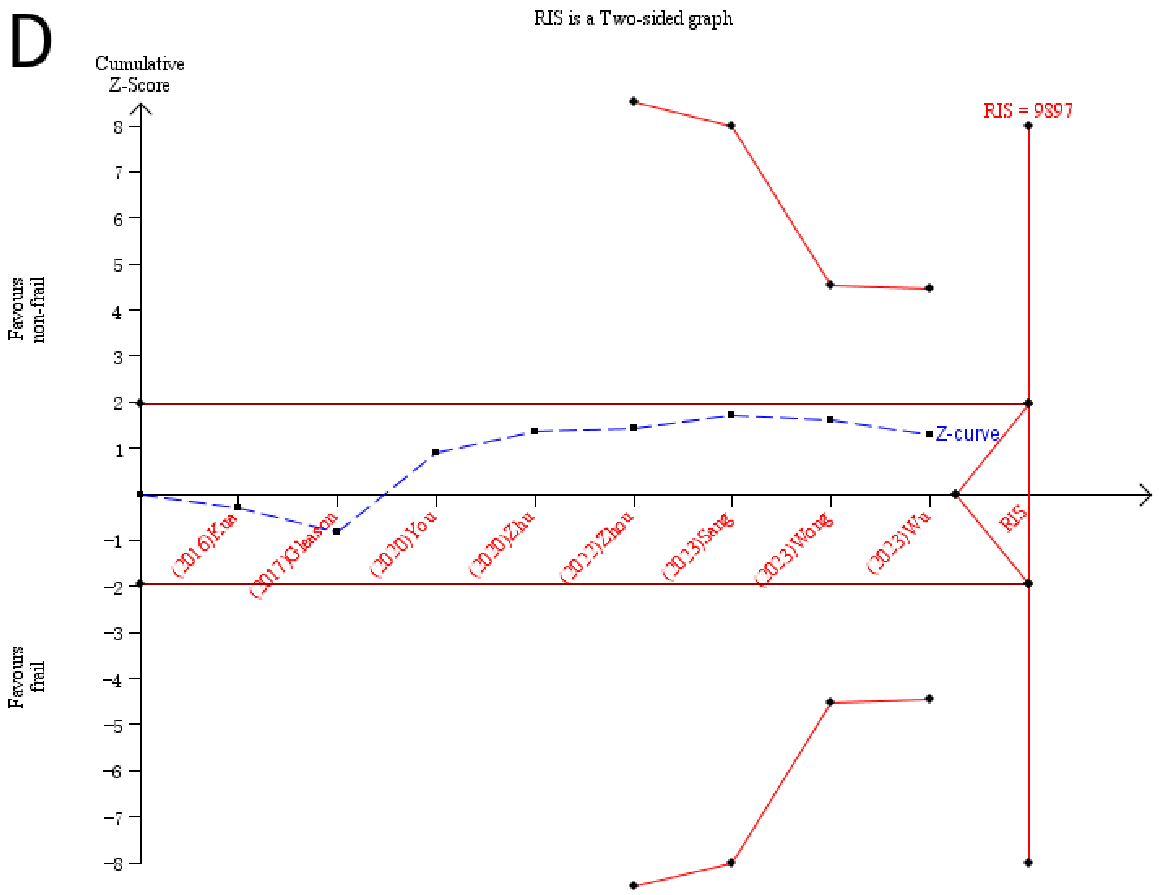

S5D Fig. Trial sequential analysis for deep venous thrombosis or pulmonary embolism predicted by frailty. The diversity-adjusted required information size of 9897 participants was based on a type of I error of 5%, a type of II error of 20%, and a control group (frail participants) proportion of 4%, with a Relative Risk Reduction of 31%.

E

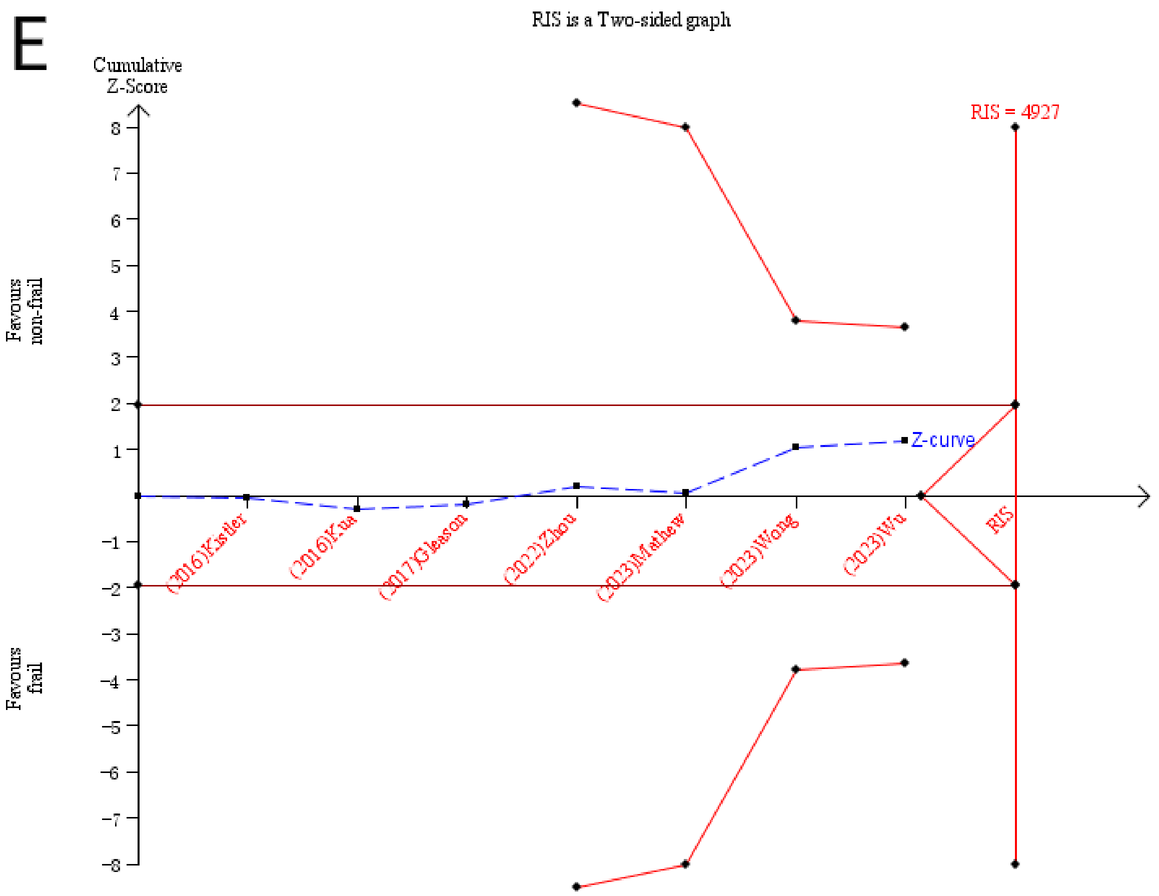

S5E Fig. Trial sequential analysis for acute kidney injury predicted by frailty. The diversity-adjusted required information size of 4927 participants was based on a type of I error of 5%, a type of II error of 20%, and a control group (frail participants) proportion of 11.5%, with a Relative Risk Reduction of 44%.

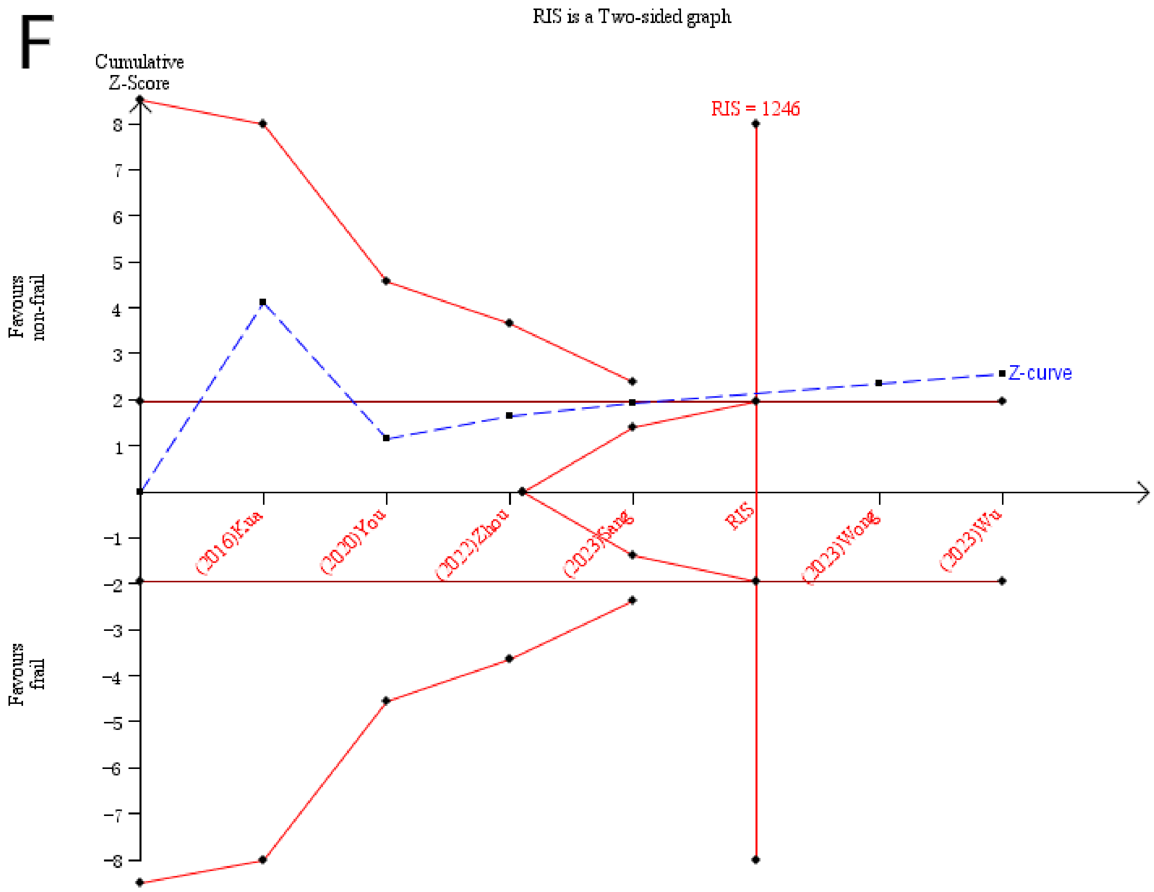

S5F Fig. Trial sequential analysis for urinary tract infection predicted by frailty. The diversity-adjusted required information size of 1246 participants was based on a type of I error of 5%, a type of II error of 20%, and a control group (frail participants) proportion of 21.1%, with a Relative Risk Reduction of 69%.
