# Supplementary material for: Association of frailty with adverse outcomes in surgically treated geriatric patients with hip fracture: A meta-analysis and trial sequential analysis
Source: PLoS One. 2024 Jun 21;19(6):e0305706. doi: 10.1371/journal.pone.0305706 (PMC11192356; doi:10.1371/journal.pone.0305706)
Supplement: S4 Table — (PDF) [file pone.0305706.s004.pdf]

S4 Table. Results of the Newcastle-Ottawa Scale quality assessment of cohort studies.

**First author, year**	**Selection**				**comparability**	**outcomes**			**Total Score**
	Representativeness of the exposed cohort (☆)	Selection of the non-exposed cohort (☆)	Ascertainment of exposure (☆)	Demonstration that outcome of interest was not present at start of study (☆)	Comparability of cohorts on the basis of the design or analysis (☆☆)	Assessment of Outcome (☆)	Was follow-up long enough for outcomes to occur (☆)	Adequacy of follow up of cohorts (☆)	
Patel 2014	☆	☆	☆	☆	☆	☆	☆	☆	8
Kistler 2016	☆	☆	☆	☆	☆☆	☆	☆	☆	9
Kua 2016	☆	☆	☆	☆	☆	☆	☆	☆	8
Choi 2017	☆	☆	☆	☆	☆☆	☆	☆	☆	9
Gleason 2017	☆	☆	☆	☆	☆☆	☆	☆	☆	9
Winters 2018	☆	☆	☆	☆	☆	☆	☆	☆	8
Jorissen 2020	☆	☆	☆	☆	☆☆	☆	☆	☆	9
Narula 2020	☆	☆	☆	☆		☆	☆	☆	7
You 2020	☆	☆	☆	☆	☆☆	☆	☆	☆	9
Zhu 2020	☆	☆	☆	☆		☆	☆	☆	7
Gandossi 2021	☆	☆	☆	☆	☆	☆	☆	☆	8
Pizzonia 2021	☆	☆	☆	☆	☆	☆	☆	☆	8
Shen 2021	☆	☆	☆	☆	☆☆	☆	☆	☆	9
Thorne 2021	☆	☆	☆	☆	☆☆	☆	☆	☆	9
Shimizu 2022	☆	☆	☆	☆	☆	☆	☆	☆	8
Zhao 2022	☆	☆	☆	☆		☆	☆	☆	7
Zhou 2022	☆	☆	☆	☆	☆	☆	☆	☆	8
Mathew 2023	☆	☆	☆	☆	☆	☆	☆	☆	8
Sang 2023	☆	☆	☆	☆	☆	☆	☆	☆	8
Wong 2023	☆	☆	☆	☆	☆	☆	☆	☆	8
Wu 2023	☆	☆	☆	☆	☆	☆	☆	☆	8
